# Privacy-Preserving Fingerprinting Against Collusion and Correlation Threats in Genomic Data

**DOI:** 10.56553/popets-2024-0098

**Published:** 2024

**Authors:** Tianxi Ji, Erman Ayday, Emre Yilmaz, Pan Li

**Affiliations:** Texas Tech University; Case Western Reserve University; University of Houston-Downtown; Case Western Reserve University

**Keywords:** genomic data, privacy, collusion, correlation attack

## Abstract

Sharing genomic databases is critical to the collaborative research in computational biology. A shared database is more informative than specific genome-wide association studies (GWAS) statistics as it enables “do-it-yourself” calculations. Genomic databases involve intellectual efforts from the curator and sensitive information of participants, thus in the course of data sharing, the curator (database owner) should be able to prevent unauthorized redistributions and protect individuals’ genomic data privacy. As it becomes increasingly common for a single database be shared with multiple recipients, the shared genomic database should also be robust against collusion attack, where multiple malicious recipients combine their individual copies to forge a pirated one with the hope that none of them can be traced back. The strong correlation among genomic entries also make the shared database vulnerable to attacks that leverage the public correlation models.

In this paper, we assess the robustness of shared genomic database under both collusion and correlation threats. To this end, we first develop a novel genomic database fingerprinting scheme, called Gen-Scope. It achieves both copyright protection (by enabling traceability) and privacy preservation (via local differential privacy) for the shared genomic databases. To defend against collusion attacks, we augment Gen-Scope with a powerful traitor tracing technique, i.e., the Tardos codes.

Via experiments using a real-world genomic database, we show that Gen-Scope achieves strong fingerprint robustness, e.g., the fingerprint cannot be compromised even if the attacker changes 45% of the entries in its received fingerprinted copy and colluders will be detected with high probability. Additionally, Gen-Scope outperforms the considered baseline methods. Under the same privacy and copyright guarantees, the accuracy of the fingerprinted genomic database obtained by Gen-Scope is around 10% higher than that achieved by the baseline, and in terms of preservations of GWAS statistics, the consistency of variant-phenotype associations can be about 20% higher. Notably, we also empirically show that Gen-Scope can identify at least one of the colluders even if malicious receipts collude after independent correlation attacks.

## 1 INTRODUCTION

In recent decades, significantly large amounts of genomic data have been generated and collected at a unprecedented rate. Among them, single-nucleotide polymorphism (i.e., SNP) data (representing point mutations in DNA) has been widely used in Genome-wide association studies (GWAS) to discover the associations between phenotypes and particular traits or human diseases. Moreover, the implementation and sharing of genomic databases, e.g., the single nucleotide polymorphism databases (**dbSNP**) [41, 42, 53] has significantly advanced the collaborative research on physical mapping, population genetics, human biology, and modern medicine [43].

**Security and Privacy Concerns.** While the benefits of collecting SNPs and constructing dbSNP are trumpeted by the computational biology community, the increased availability of such data has raised concerns about the data owners’ copyright and the data contributors’ privacy. Thus, an owner of dbSNP will only share its data to authorized recipients, e.g., service providers (SPs) like hospitals and research institutions after data use agreements and also want to prevent illegal redistribution of data. Most importantly, when data leakage happens, genomic database owner needs to be able to collect evidence to accuse the party (or parties) who should be responsible for the leakage. For example, in commercial genetic testing, such as 23andme [1], companies providing genetic testing services need to collect and store genomic data from other resources. Such genomic data can be used for research purposes for the benefits of the participating customers, but must be protected from unauthorized redistribution.

On the other hand, genomic data, such as SNP and nucleobases, contains sensitive features that can be used to identify individuals (via forensics), connect to other family members (via kinship), and infer individuals’ health condition (associating SNPs with diseases) [36]. For example, information about the number of minor alleles (alleles/nucleotides that are observed less frequently in DNA) in an individual can be used to identify that person’s identity through genetic profiling^1^, which is controversial and raises ethical, legal, and privacy concerns. Hence, the data curator is also obligated to protect the privacy of the individuals (data contributors). It is noteworthy that the General Data Protection Regulation (GDPR) lists genetic data as “special categories of personal data” that is subject to organizational and technical safeguards [39].

### 1.1 Entire Database v.s. Summary Statistics

We focus on the sharing of the entire dbSNP instead of releasing specific GWAS statistics (e.g., correlation between SNP pairs or allele frequencies) under differential privacy. This is because in a typical GWAS process, the researchers do not know in advance which SNP pairs to use and what types of statistical tests to query [40]. Usually, the number of significant SNPs associated with specific diseases and the pairs of correlated SNP entries are the results of GWAS process, not the input [28].

Thus, in this work, we are motivated to first develop a feasible genomic database sharing scheme to provide researchers access to genomic data for the purposes of collaborative research and “**do-it-yourself**” calculations, which provides more freedom than simply allowing computations on a server owned by genomic database owner. To address the above security and privacy concerns, the developed scheme should have both copyright protection and privacy preservation guarantees for the shared genomic databases.

### 1.2 Our Solution

In the literature, quite a few works have attempted to address the problems of protecting the copyright and privacy of dbSNP in isolation. In particular, in order to protect copyright and deter illegal redistribution, a series of genome watermarking/fingerprinting schemes have been developed [5, 24, 37, 55]. To protect the privacy of the individuals in genomic databases, both cryptographic techniques [4, 6, 58] and statistical approaches (via differential privacy) [21, 26, 28, 50, 56, 57] have been proposed. However, encrypted genomic data only allows a limited number of operations and usually requires high computation costs. Thus, differential privacy-based genomic data sharing has been widely adopted.

To achieve both copyright protection and privacy preservation for genomic databases, a straightforward two-step approach is to insert fingerprint into a differential-privately sanitized dbSNP. However, this significantly reduces the utility of the final dbSNP (as will be empirically shown in Section 8), because it requires adding separate noises to achieve the two guarantees; first adding noise to attain privacy guarantee, and then adding additional noise (via fingerprinting) to obtain copyright guarantee.

In this paper, we propose **Gen-Scope**, which shares **gen**omic databases and **s**imultaneously achieve **co**pyright protection and **p**rivacy pres**e**rvation via one-shot noise (fingerprint) insertion. In Gen-Scope, the inserted fingerprint can also be used to protect the privacy of the genomic data. The key idea is to leverage the intrinsic randomness of fingerprint insertion and transform it into a provable privacy guarantee [23]. In particular, we first observe that fingerprint insertion essentially flips each bit of a SNP data randomly, and this leads to the value of that SNP being changed with certain probability, then from which, we can derive the privacy guarantee in the form of local differential privacy (LDP) [15]. Since Gen-Scope only inserts noise once, the final dbSNP has high utility (measured in terms of the accuracy of dbSNP and GWAS statistics).

Part of the Gen-Scope is adapted from previous works on relational databases fingerprinting [23–25, 27]. However, both [24] and [23] are only robust against random bit flipping attack, subset attack, superset attack, and correlation attacks [27]; the inserted fingerprints may still be compromised by collusion attack (possibly after a few rounds of correlation attacks). To address this issue, we improve [23, 24] by incorporating the Tardos code [48], which is one of the most powerful techniques to fight against collusion attacks by identifying the colluders with very high probability.

Furthermore, [24] does not consider the privacy of the shared database, whereas, in this work, our main goal is to simultaneously achieve LDP and robust fingerprinting with high database utility. On the other hand, the privacy guarantee developed in [23] cannot be directly cast into LDP, because LDP requires that after perturbation, each data entry has non-zero probability of taking any other values in the input domain, whereas [23] does not allow the original data value to be modified to certain values from the domain.

**Contributions.** This work is the first to achieve genomic database fingerprinting with LDP guarantee. The proposed Gen-Scope can also be augmented to defend against the collusion attacks launched by allied attackers after correlation attacks. In particular,

- We derive a closed-form expression which connects the percentage of fingerprinted bits (γ) with the robustness against random bit flipping attack and collusion attack. We also empirically investigate the robustness against correlation attacks.
- We analyze the required fraction of changed SNP entries for Gen-Scope and the two-stage approach (differentially private perturbation followed by fingerprinting) to achieve the required privacy and copyright guarantees.
- Experiment results show that, under the same guarantees of copyright protection and privacy preservation, Gen-Scope results in dbSNP with higher utility than the two-step approach. For example, the accuracy of the fingerprinted genomic database obtained by Gen-Scope can be 10% higher than that achieved by the naïve two-stage approach, and in terms of preservation of GWAS statistics, the consistency of SNP-phenotype associations can be 20% higher. When the shared dbSNP is compromised by correlation attacks followed by collusion attack, Gen-Scope can still identify at least one of the colluders.

Gen-Scope helps facilitate the progress of collaborative genomic research by relieving the tension between the utility of genomic databases and the privacy of participants as well as the rights of the genomic database owner.

**Roadmap.** We review related works in Section 2. Preliminaries on database fingerprinting and genomics are reviewed in Section 3. In Section 4, we describe the system and threat models, and the evaluation metrics. Section 5 introduces Gen-Scope, and Section 6 discusses how to improve it to defend against the collusion attack. In Section 7, we derive a closed-form expression connecting the density of fingerprinted bits and the corresponding robustness and also analyze the required amount of modification on SNP entries. In Section 8, we compare Gen-Scope with the two-step approach. Finally, Section 9 concludes the paper.

## 2 RELATED WORK

Fingerprinting techniques are originally proposed to prevent illegal redistribution of multimedia, e.g., images [17], audio [7], videos [47], and text documents [11]. The first work that applies unique fingerprinting (i.e., watermarking) to relational database is [2], which modifies insignificant bits of data entries to preserve the utility of the database. Different from fingerprinting, in watermarking all service providers (SPs) receive the same watermarked copy, so it is not feasible to trace the source of data leakage.

Afterwards, some works using [2] as the building block have been proposed [18, 32, 33]. For example, [32] allows that the inserted fingerprint can be arbitrary bit-strings. [27] develops fingerprinting scheme that can defend attacks that leverage the correlations among data records. Most recently, a database fingerprinting scheme with provable privacy guarantees is developed in [23]. However, the authors in [23] term their privacy guarantee as entry-level differential privacy, which is unfamiliar to genetics practitioners. In particular, in entry-level DP, only limited number of insignificant bits will be modified, thus the modified data entries cannot span the original input domain. Whereas, in LDP, the domain of perturbed data entries is identical to the original data domain, hence, all bits should be subject to equal probability of being modified.

The first genomic fingerprinting scheme was proposed in [5], which shares personal genomic sequential data by jointly considering collusion attack and data correlation. Then, [55] develops a probabilistic fingerprinting scheme by considering the conditional probabilities between SNPs of a single individual. [37] develops a watermarking scheme for sequential SNP data based on belief propagation which considers privacy and watermark robustness. However, these works all focus on the genomic data (SNPs) of a single individual, rather than a genomic database, i.e., a collection of individuals’ SNP record. Very recently, [24] proposes the first fingerprinting scheme that can handle collections of genomic sequences by extending [27, 32]. In Table 1, we summarize the differences between existing works and this paper.

This work is different from all the previously mentioned works on genomic data fingerprinting, because it is the first to investigate all 3 problems together, i.e., (i) fingerprinting an entire genomic database (instead of single genomic record), (ii) achieving copyright protection and privacy preservation via one-shot steganographic mark insertion, and (iii) defending against both collusion attacks and correlation attacks.

## 3 PRELIMINARIES

In this section, we provide background information for database fingerprinting and genomics in general.

### 3.1 Database Fingerprinting Techniques

Database fingerprinting schemes are steganography techniques that randomly change selected data entries with certain probability. The modified values of the selected entries in a given database are determined by a unique binary bit-string customized for each database recipient. The value of the binary bit-string (i.e., the fingerprint/steganographic mark of the recipient) is obtained by a message authentication code (MAC) involving a cryptographic hash function, a secret cryptographic key of the database owner, and the identity of the recipient. The process of modifying data points based on the fingerprint is called fingerprint insertion. Since the fingerprints are hard to be detected or compromised, a malicious recipient will be held responsible if it leaks its received database.

### 3.2 Genomic and GWAS Background

#### 3.2.1 Single Nucleotide Polymorphism.

Double stranded DNA molecules in the human genome are composed of two complementary polymer chains, each containing nucleotides (i.e., A, C, G, T). Although most of the DNA sequence is similar across the entire human population, roughly 0.5% of an individual’s DNA (which equates to millions of nucleotides) differs from the reference genome [30, Chapter 2]. The most common type of DNA variation is a Single Nucleotide Polymorphism (SNP). Each person has approximately 4 million SNPs. A SNP is the mutation at a single nucleotide at a particular loci of the genome. For each SNP, there are two types of nucleotides (or alleles), i.e., major allele (the allele that is observed with a high frequency) and minor allele (the allele that is observed with a low frequency). Each SNP includes two nucleotides, one inherited from each parent. As a result, biologist represents a SNP using the number of minor alleles (0, 1, or 2). Below is a toy example on the SNP from three individuals [54].

**Example 1**. Suppose we consider the SNP at position 1000 on the Chromosome 1 (the largest human chromosome). At this position, individuals may have different nucleotides. The reference genome is a ‘C’ nucleotide at this position. However, in some individuals, there could be a mutation where instead of ‘C’, they have an ‘A’ nucleotide. Thus, for this SNP at position 1000 on Chromosome 1, the major allele is ‘C’, which is the nucleotide observed with high frequency in the population. The minor allele is ‘A’, observed with lower frequency.

Each individual would have two alleles at this SNP position, one inherited from each parent. If Alice has inherited ‘C’ from one parent and ‘C’ from the other parent, indicating Alice has 0 minor alleles at position 1000. If Bob has inherited ‘C’ from one parent and ‘A’ from the other parent, indicating he has 1 minor allele at position 1000. If Charlie has inherited ‘A’ from both parents, indicating he has 2 minor alleles at this position. As a result, the SNP value at position 1000 for Alice, Bob, and Charlie is 0,1, and 2, respectively.

This paper considers the genomic database, which is a collection of SNPs of a certain population, i.e., dbSNP [53]. In dbSNP, each row corresponds to the SNP sequence of an individual. Suppose there are N individuals and each has P SNPs, then, the dbSNP is represented as $\text{R}\in \{0,1,2{\}}^{N\times P}$.

#### 3.2.2 Genome-wide Association Studies.

The genetic makeup of an organism is referred to as its genotype, while the observable traits it exhibits are known as its phenotype. For instance, the ability to roll one’s tongue represents a phenotype, while the underlying genetic factors influencing tongue rolling ability constitute the genotype. The genotype is inherited from an organism’s parents, while the phenotype is not directly inherited. Instead, phenotype is shaped by a combination of factors including the genotype, epigenetic modifications, environment, and etc. Establishing universally accepted taxonomy or encoding standards for phenotype data remains a challenge due to its multifaceted nature. GWAS [51] focuses studying the associations between SNP and phenotype (e.g., the characteristics of being able to roll one’s tongue). For example, a GWAS on tongue rolling ability will investigate the genetic variant (SNPs) whose genotypes are associated with the ability to roll one’s tongue.

Individuals participating in GWAS are categorized into two groups: those exhibiting a specific trait, such as the ability to roll one’s tongue, are grouped as cases, while those lacking such a trait are grouped as controls, see, e.g., [35]. GWAS examines the genomes of participants in both case and control groups. If a particular type of genetic variant, such as a SNP, is found to occur more frequently in individuals with the trait (i.e., in the case group), it is deemed to be associated with the trait. The most popular statistical method applied in GWAS is the p-value measurement [19, 44]. In particular, SNPs with are considered to have strong associations with the phenotype if the corresponding p-value is low. More details are deferred to Section 4.2.

Recently GWAS have revealed that a patient’s risk for specific diseases can be partially predicted based on their SNPs [36]. As a result, the leakage of SNPs can pose a significant threat to an individual’s privacy, and the sharing of relational database consisting of individuals’ SNPs should be regulated with copyright protections.

## 4 SYSTEM, THREATS, AND METRICS

In this section, we first present an overview of the proposed Gen-Scope, and then discuss its properties, i.e., the guarantees on copyright protection and privacy preservation against various threat models. Lastly, we provide the utility metrics of the shared dbSNP.

### 4.1 Gen-Scope Overview

We consider a database owner (Alice) with a dbSNP represented using R. Each SNP (i.e., the entry of the database) is represented by the number of its minor alleles as 0, 1, or 2, and can be encoded as “00”, “01”, or “10”, respectively.

**Figure 1:**
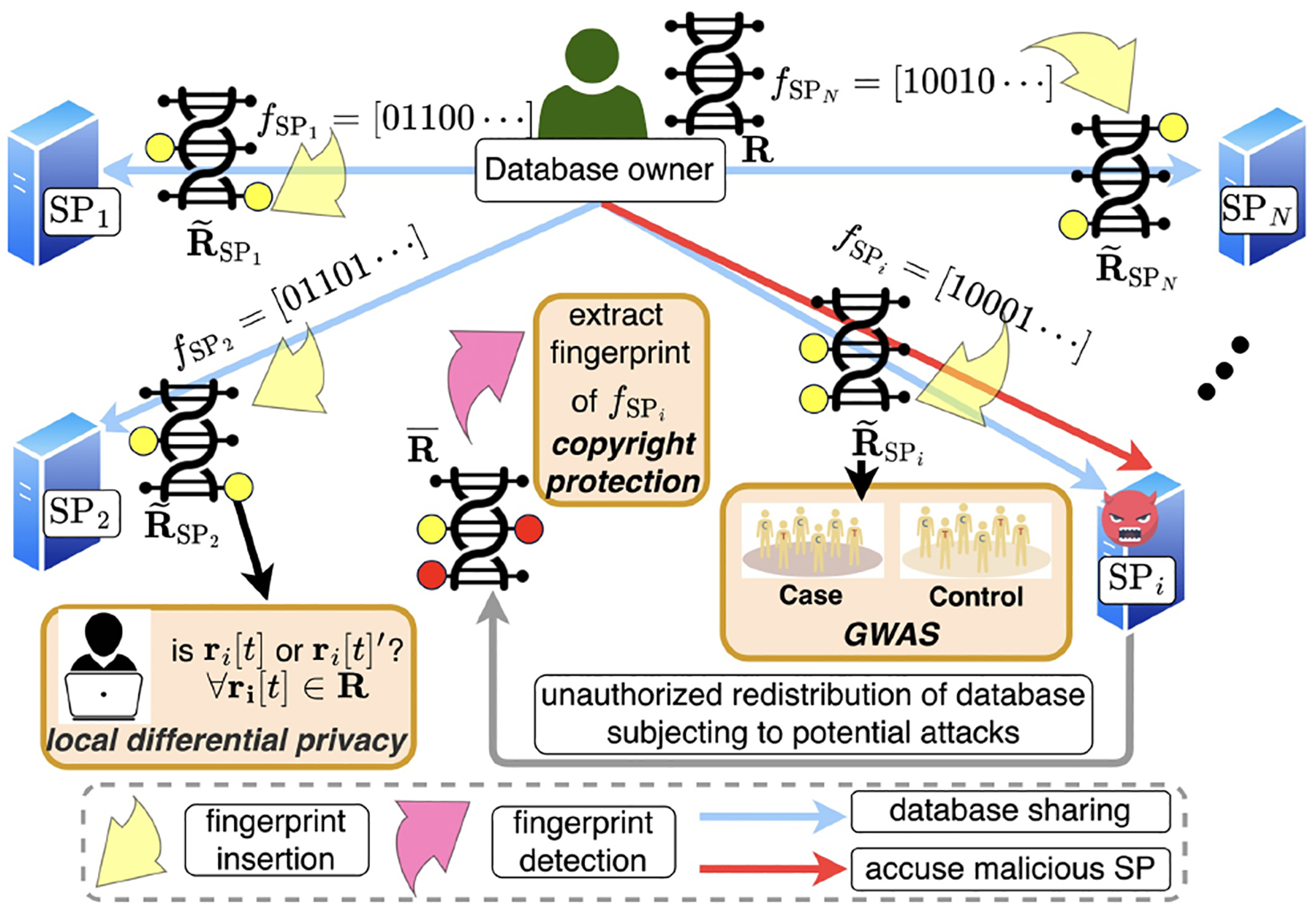
[Adapted from [23]] Alice adds a unique fingerprint in each copy of her dbSNP when sharing. The fingerprint changes entries at different locations (the yellow dots) in R. She can identify the malicious SP who pirates and redistributes her dbSNP using a distorted fingerprint. All shared dbSNP copies achieve LDP and fingerprint robustness.

We show the overview of the system model in Figure 1 (adapted from the general relational database sharing in [23]). Alice wants to share the genomic database R with N SPs. To prevent unauthorized redistribution of the database by a malicious SP, Alice embeds unique fingerprints in all shared copies of the dbSNP. The fingerprint essentially changes different entries in R at different SNP positions (indicated by the yellow dots). The fingerprint (a binary bit-string) generated for the ith SP $\left({\text{SP}}_{i}\right)$ is $f_{{\text{SP}}_{i}}$, and the dbSNP received by ${\text{SP}}_{i}$ is ${\tilde{\text{R}}}_{{\text{SP}}_{i}}$. Both $f_{{\text{SP}}_{i}}$ and ${\tilde{\text{R}}}_{i}$ are obtained using the proposed scheme (see Section 5). In Figure 1, if ${\text{SP}}_{i}$ forges a pirated dbSNP, i.e., $\bar{\text{R}}$, by changing some values (indicated by the red dots) in its received copy, i.e., ${\tilde{\text{R}}}_{{\text{SP}}_{i}}$, Alice is able to accuse ${\text{SP}}_{i}$ for data leakage with high probability by extracting $f_{{\text{SP}}_{i}}$ from $\bar{\text{R}}$. In addition to the copyright protection, Alice also preserves the privacy of SNP data and maintain high database utility.

#### 4.1.1 Properties of Gen-Scope.

In general, a genomic database recipient (SP) can be any of the following: (1) an honest party who will use the received dbSNP to perform GWAS, (2) an attacker who wants to make illegal profits by changing some entries in its received dbSNP and making pirate copies of it, or (3) a curious party who tries to infer the original SNP values. Thus, our proposed Gen-Scope is designed to achieve the following properties

- (i) high utility (measured in terms database accuracy and consistency of SNP-phenotype association) for the fingerprinted dbSNP in order to support accurate GWAS,
- (ii) copyright protection to discourage illegal redistribution, i.e., to successfully extract a malicious SP’s fingerprint when Alice identifies a pirated version of the released dbSNP (even if the malicious SP tries to distort the fingerprint in its received dbSNP),
- (iii) local differential privacy guarantee against attributes inference attacks, i.e., a data analyst cannot distinguish between ${\text{r}}_{i}[t]$ and ${\text{r}}_{i}[t{]}^{\prime }$ by using its received copy of dbSNP.

Although (ii) and (iii) are different properties, they can be achieved at the same time (by leveraging the intrinsic randomness during fingerprint insertion), however, at the cost of (i). Thus, in practice, the database owner needs to strike a balance between the requirements of (ii) (iii) and (i). In this paper, we assume that Alice is benign (i.e., she will not modify its own dbSNP to frame any SP).

#### 4.1.2 Threats to Gen-Scope.

The objectives of malicious SPs are

- (a) illegally redistribute received dbSNP (i.e., make pirated copies by launching various attacks targeting the inserted fingerprint bit-string) without being accused by Alice, **and**/**or** launch inference attack aiming to recover the original values of SNPs in its received dbSNP,
- (b) preserve database utility to gain illegal profit.

Malicious SPs will introduce extra utility loss while distorting the fingerprint in received dbSNPs. That is (a) and (b) are also conflicting objectives. We consider that all malicious SPs are rational (i.e., they will not over-distort a dbSNP, otherwise they cannot make illegal profit out of a pirated copy with poor utility). Thus, a rational SP will try to get away with making pirated copies of the dbSNP by changing as few SNP values as possible.

Next, we discuss the threats to copyright and privacy separately.

**Threats to copyright**. In this paper, we mainly focus on the following attacks targeting on the inserted fingerprints.

- Random Bit Flipping Attack [2]. To pirate a dbSNP, a malicious SP can select random bit positions in its received copy of the genomic database and flip their bit values, e.g., a SNP value 0 (“00”) becomes 1 (“01”) after the attack. As will be shown in Section 8, Gen-Scope is robust against this threat even if the attacker flips more than 45% of the bits in its received copy.
- Collusion Attack [8, 9, 38]. Via collusion, two or more malicious SPs combine their individual versions of fingerprinted dbSNP to forge a pirated copy in hope of that none of them can be traced back. In Section 6, we will show that by adopting the Tadros codes [48], Gen-Scope can be collusion-resistant.
- Correlation Attacks [24]. By modifying the SNP values a dbSNP, the inserted fingerprints will make the correlations between genome sequence deviate from the original correlation models. Thus, an attacker can compare the publicly available correlation models (e.g., Mendel’s law and/or linkage disequilibrium) with the empirical correlations obtained from fingerprinted dbSNP to infer and compromise the fingerprinted entries. In Section 8.4, we will show that Gen-Scope is also robust against correlation attacks.

**Threats to privacy.** Malicious SPs may also try to infer the original values of specific SNPs of individuals to compromise the privacy of sensitive information about individuals, e.g., the predisposition to diseases and family relationships [57]. In Gen-Scope, by leveraging the randomness in fingerprinting, we achieve plausible deniability for the individuals.

In this paper, we only consider the attribute inference attack in privacy threat due to the constraint of the relational model of the genomic database, where each genomic data record can be uniquely referred to by an **immutable** primary key (see Definition 1). It is a hard requirement that the primary keys (i.e., pseudo IDs of data records) are immutable in relational databases, because they also serve as the foreign keys to link between different databases in DBMS (database management system). Such property is leveraged in all database fingerprinting schemes, e.g., [2, 24, 27]. As a direct consequence, it is no secret whether an individual’s genomic record is present in a dbSNP or not due to the uniqueness and immutability of the primary keys. Hence, the membership inference attack against differential privacy becomes an ill-posted problem in the scenario of fingerprinting a relational database (please refer to [23, Section III] for more elaboration).

### 4.2 Fingerprint Robustness and Utility Metrics

The primary goal of a malicious SP is to distort the fingerprint in its received dbSNP before redistribution to avoid being accused. Similar to [23–25, 27], we use the percentage of compromised fingerprint bits, i.e., ${\text{Per}}_{\text{cmp}}$, to measure the robustness of the fingerprint scheme.

$${\text{Per}}_{\text{cmp}}=\frac{1}{L}\times \sum_{l=1}^{L}1\left\{f\left(l\right)\neq \overset{‾}{f}\left(l\right)\right\},$$

where 1{⋅} is the indicator function, L is the length of the fingerprint bit-string, $\overset{‾}{f}$ is the extracted fingerprint bit-string from $\bar{\text{R}}$ (i.e., a pirated dbSNP), and $f(l)(\text{or}\;\overset{‾}{f}(l))$ is the lth bit in f (or $\overset{‾}{f}$). In [24, 27], the authors have shown that if ${\text{Per}}_{\text{cmp}}>50\%$, a malicious SP can cause the database owner to accuse other innocent SPs who also received the databases. In this paper, we only focus on ${\text{Per}}_{\text{cmp}}$, because other robustness metrics (e.g., the accusable ranking of a malicious SP) can be derived from ${\text{Per}}_{\text{cmp}}$ [27].

Fingerprinting naturally changes the values of SNPs, and hence degrades the utility of the dbSNP. Similar to [24], we also quantify the utility of a fingerprinted dbSNP using the following metrics.

**Accuracy of the database, i.e.,** *Acc*. This calculates the percentage of matched SNP entries between the original dbSNP and the fingerprinted copy (or the pirated copy). In Section 7, we will present close-form relationships between accuracy (100% minus percentage of changed entries), fingerprinting robustness, and privacy guarantee.

**Consistency of SNP-phenotype association.** GWAS quantifies the associations between a phenotype and each SNP in a dbSNP using a p-value with a confidence level of 95% [19, 44]. SNPs with low p-values (typically less than 0.05) are considered to have strong associations with the phenotype (i.e., the association is very unlikely to be due to chance). Generally, a larger utility loss in terms of accuracy degradation will lead to less accurate SNP-phenotype association. To evaluate the p-value of each SNP in a dbSNP, we first randomly divide the database into non-overlapping case (denoted as S) and control (denoted as C) groups, and then follow the steps listed in (1) to perform the calculations.

(1) $$\text{OR}=\frac{C_{0}\left(S_{1}+S_{2}\right)}{S_{0}\left(C_{1}+C_{2}\right)},\;\text{StdErr}(\text{ln}(\text{OR}))=\sqrt{\frac{1}{S_{1}+S_{2}}+\frac{1}{S_{0}}+\frac{1}{C_{1}+C_{2}}+\frac{1}{C_{0}}},\;z=\frac{\text{ln}(\text{OR})}{\text{StdErr}(\text{ln}(\text{OR}))},\;p=\Psi (-z)+1-\Psi (z).$$

Here OR is the odd ratio, $S_{0},\;S_{1}$, and $S_{2}$ (or $C_{0},\;C_{1}$, and $C_{2}$) are the numbers representing a specific SNP taking a value of 0, 1, and 2 in the case (or control) group. StdErr(ln(OR)) denotes the standard error of the logarithm of the odd ratio, and z is the standard normal deviation (i.e., z-value). Finally, the p-value is the area (probability) of the normal distribution that falls outside ±z, and it can be obtained using Ψ(⋅); the cumulative distribution function of the standard normal distribution.

To evaluate the GWAS utility, we identify the top-K percentage SNPs (the K percentage SNPs with the lowest p-values) from the original (non-fingerprinted) dbSNP (R). Then, we check the fraction such SNPs being preserved (i.e., remains to be the top-K percentage SNPs) after fingerprinting or various attacks. Since GWAS utility depends on the dbSNP and the partition of case/control group, we will empirically evaluate it in Section 8.2.2.

## 5 THE PROPOSED GEN-SCOPE SCHEME

In this section, we first review the relational model of dbSNP. Then, we discuss how to leverage the randomness of fingerprinting to preserve privacy in dbSNP sharing; we derive a sufficient condition connecting fingerprinting to ϵ-LDP. Next, we present Gen-Scope that complies with the sufficient condition and enables copyright protection and privacy-preservation simultaneously.

### 5.1 Relational dbSNP and Privacy Model

Definition 1 (Relational model of dbSNP [12, 24]). *A dbSNP* (R) *is a collection of* T-tuples. Each of these tuples represents the SNP sequence of a specific individual. Each SNP sequence is associated with a primary key, i.e., a pseudo-identifier used to uniquely identify that individual. The SNP sequence of the ith individual is denoted as ${\text{r}}_{i}$ *and the primary key of that individual is* ${\text{r}}_{i}$.*PmyKey*.

It is noteworthy that in DBMS (database management system), the primary keys must be immutable [3, 13, 20] to support database operations, like union, intersection, and update. Primary keys also serve as foreign keys (a column that creates a relationship between two tables in DBMS). Thus, updating a primary key can mess up many other tables or rows in the DBMS. As a result, when fingerprinting a relational genomic database, the primary keys should not be changed if a dbSNP is fingerprinted or pirated [2, 31, 32]. In other words, the fingerprint bit-string only changes the attributes (i.e., the SNP values) of individuals and keeps their pseudo-identifiers intact. If a malicious SP destroys all primary keys when redistributing a dbSNP, such dbSNP will be considered to have no utility, because its linkability to other genomic databases in DBMS is lost, thus, can hardly support a wide range of GWAS or biomedical research.

Definition 2 (ϵ-LDP [14]). *A randomized mechanism* ℳ *with domain* 𝒟 *satisfies* ϵ-LDP *if the following holds for all pairs of single data entry* x *and* $x^{\prime }$,

$$\text{Pr}\left[ℳ\left(x\right)=y\in \text{Range}\left(ℳ\right)\right]\leq e^{\epsilon }\text{Pr}\left[ℳ\left(x^{\prime }\right)=y\in \text{Range}\left(ℳ\right)\right],$$

*where Range* (ℳ) *is the set of all possible outputs for a data entry*.

## 5.2 Connecting Fingerprinting to Privacy

Similar to all database fingerprinting schemes [2, 18, 25, 27, 29, 32, 33], which change randomly selected bits of encoded data using a certain probability, Gen-Scope also flips each of the two bits of a SNP with probability p(p<0.5). The change to bits (i.e., flipping) is performed by conducting an exclusive or (XOR) operation between that bit and a Bernoulli random variable B~Bernoulli(p). The collections of selected SNP bits vary for different SPs, and their fingerprinted values (the results after the XOR operation) are decided by the unique fingerprint bit-strings of the SPs. Thus, database fingerprinting is a randomized mechanism, which is endowed with certain level of privacy guarantee. The following theorem shows that by calibrating the probability (p), fingerprinting is able to achieve LDP for each SNP entry.

Theorem 1. *Given a dbSNP* R, *a fingerprinting scheme (denoted as* ℳ), *that conducts the XOR operation between a bit of SNP and a Bernoulli random variable* B~Bernoulli(p), *is said to achieve* ϵ-*local differential privacy with respect to each SNP, if* $p\geq \frac{1}{e^{\epsilon /2}+1}$.

Theorem 1 can be proved by adapting the steps developed in [23]. Please refer to Appendix A.1 for the detailed proof. It is noteworthy that the achieved DP guarantee in Theorem 1 is different with the one in [23], because [23] only allows limited insignificant bits to be modified by the mark bits, thus, after perturbation, the output data entries cannot span the original input data domain. Whereas, since SNP data can be fully characterized by two bits, and the marked bits are applied to all bits of the SNP data, LDP can be achieved while fingerprinting the genomic relational database.

## 5.3 dbSNP Fingerprinting meeting ϵ-LDP

This section provides an exposition of our proposed Gen-Scope, which satisfies the sufficient condition developed in Theorem 1; Gen-Scope fingerprints dbSNP with ϵ-LDP guarantee.

### 5.3.1 Fingerprint Insertion.

First, we collect all bits in R in a set $𝒫:𝒫=\left\{{\text{r}}_{i}[t,k]|i\in [1,N],t\in [1,T],k\in [1,2]\right\}$, where N is the number of individuals in R, and T is the total number of SNPs for each individual. When the database owner (Alice) wants to share a fingerprinted copy of R with an SP which has a publicly known ID denoted as ID, she generates the unique fingerprint for this SP via f=HMAC(Y∣ID), which is a message authentication code (MAC) involving a cryptographic hash function and a secret cryptographic key (Y is the secret key of Alice and | represents the concatenation operator).

Alice also has a cryptographic pseudorandom sequence generator 𝒰, which selects the SNP entries and their bits, and determines the mask bit x and fingerprint bit f (which is an element of the fingerprint bit-string **f**) to obtain the Bernoulli random variable (i.e., B=x⊕f). To be more specific, for each ${\text{r}}_{i}[t,k]$ (the kth to last bit of the tth SNP of individual i) in 𝒫, Alice sets the initial seed as $s=\left\{Y|{\text{r}}_{i}.\text{PmyKey}|t|k\right\}$. If ${𝒰}_{1}(s)\;\text{mod}\;\left\lfloor \frac{1}{2p}\right\rfloor =0\left(p>\frac{1}{e^{\epsilon /2}+1}\right)$, then ${\text{r}}_{i}[t,k]$ is fingerprinted using the following steps. The steps to generate a fingerprinted dbSNP $\tilde{\text{R}}$ for SP, ID, is summarized in Algorithm 1. In particular, the subroutine of fingerprinting a specific SNP bit is shown in (2).


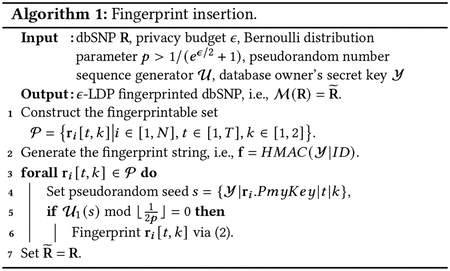

(2) $$\text{Set}\;\text{mask}\;\text{bit}\;x=0,\;\text{if}\;{𝒰}_{2}(s)\;\text{is}\;\text{even};\;\text{otherwise}\;x=1.\;\text{Set}\;\text{fingerprint}\;\text{index}\;l={𝒰}_{3}(s)\;\text{mod}\;L.\;\text{Let}\;\text{fingerprint}\;\text{bit}\;f=\text{f}(l).\;\text{Obtain}\;\text{mark}\;\text{bit}\;\;B=x⊕f.\;\text{Set}\;{\text{r}}_{i}\left[t,k\right]={\text{r}}_{i}\left[t,k\right]⊕B.$$

Now, we arrive at the following LDP guarantee on the fingerprinting scheme on dbSNP. This privacy guarantee is a specialization of the entry-level privacy guarantee on general relational database. Its proof can be adapted from [23] and is shown in Appendix A.2.

Theorem 2. Algorithm 1 *is* ϵ-*local differentially-private*.

**Post-processing.** After fingerprinting the genomic database (R), some entries may have values that are outside the domain of the SNP (i.e., {0,1,2}). Thus, we also need to post-process the resulting database (ℳ(R)) to eliminate entries that are not in the original domain. Otherwise, the database recipient can understand that these entries are changed due to fingerprinting. Due to the post-processing immunity property of DP, there is no privacy degradation in this step. Even though the post-processing may alter some fingerprinted entries, it has negligible impact on the fingerprint robustness, because it only changes a small fraction of fingerprinted entries, and in the fingerprint extraction phase, we determine the value of each bit in the fingerprint by counting how many times it has been extracted as 1 or 0 followed by majority voting, i.e., each bit of the fingerprint is recovered by the majority voting on the positions marked by this fingerprint bit (i.e., Line 8 in Algorithm 2).

Generally, post-processing steps are able to make a fingerprinted database meet the domain requirements so as to achieve better utility in downstream applications. In [27], the authors show that post-processing steps can make a fingerprinted database preserve the column- and row-wise data correlations and the covariance matrix of the database, which are frequently utilized to establish predictive models, e.g., regression and probability fitting.

#### 5.3.2 Fingerprint Extraction.

When Alice observes a leaked (or pirated) dbSNP denoted as $\bar{\text{R}}$, she will try to identify the traitor (malicious SP) by extracting the fingerprint from $\bar{\text{R}}$ and comparing it with the fingerprints of SPs who have received her database.


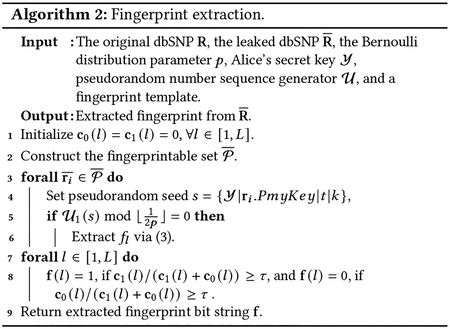


We show the fingerprint extraction procedure in Algorithm 2. In particular, Alice first initiates a fingerprint template $\left(f_{1},\cdots ,f_{L}\right)=(?,?,\cdots ,?)$. Here, “?” means that the fingerprint bit at that position remains to be determined. Then, Alice locates the positions of the fingerprinted bits exactly as in Algorithm 1, and fills in each “?” using majority voting. To be more precise, she first constructs the fingerprintable sets $\bar{𝒫}$ from $\bar{\text{R}}$, i.e., $\bar{𝒫}=\left\{{\bar{\text{r}}}_{i}[t,k]|i\in \right.[1,\overset{‾}{N}],t\in [1,T],k\in [1,2]\}$, where $\overset{‾}{N}$ is the number of records in $\bar{\text{R}}$. Note that, in general, $\overset{‾}{N}\neq N$, because a malicious SP may conduct the subset attack to remove some SNP sequences from the received dbSNP before leaking it. Then, Alice selects the same bit positions, mask bit x, fingerprint index l using the pseudorandom seed $s=\left\{Y|{\text{r}}_{i}\right..\text{PmyKey}\left.|t|k\right\}$, and recover (extract) $f_{l}$ using steps in (3), which is exactly the reverse of (2).

(3) $$\text{Set}\;\text{mask}\;\text{bit}\;x=0,\;\text{if}\;{𝒰}_{2}(s)\;\text{is}\;\text{even};\;\text{else}\;x=1.\;\text{Set}\;\text{fingerprint}\;\text{index}\;l={𝒰}_{3}(s)\;\text{mod}\;L.\;\text{Recover}\;\text{mark}\;\text{bit}\;B=\bar{{\text{r}}_{i}}[t,k]⊕{\text{r}}_{i}[t,k].\;\text{Recover}\;\text{fingerprint}\;\text{bit}\;f_{l}=x⊕B$$

Since the value of $f_{l}$ may be changed due to various attacks, Alice will maintain and update two counting arrays ${\text{c}}_{0}$ and ${\text{c}}_{1}$, where ${\text{c}}_{0}(l)$ and ${\text{c}}_{1}(l)$ record the number of times $f_{l}$ is recovered as 0 and 1, respectively. Finally, Alice sets f(l)=1 if ${\text{c}}_{1}(l)/\left({\text{c}}_{1}(l)+{\text{c}}_{0}(l)\right)\geq \tau$, and f(l)=0 if ${\text{c}}_{0}(l)/\left({\text{c}}_{1}(l)+{\text{c}}_{0}(l)\right)\geq \tau$, otherwise she keeps f(l)=? (i.e., this fingerprint bit cannot be determined due to low confidence), where τ∈(0.5,1] is the parameter that quantifies Alice’s confidence in the fingerprint recovery phase.

## 6 AUGMENTING GEN-SCOPE AGAINST COLLUSION ATTACK USING TARDOS CODE

When fingerprinted copies of dbSNP are shared with multiple SPs, two or more malicious SPs can combine their individual versions of dbSNP to forge a pirated copy in hope that none of them can be traced back, which is known as the collusion attack [8, 48].

In the literature, several works have proposed collusion-resistant fingerprinting schemes for relational databases, e.g., Boneh-Shaw (BS) codes [8, 9] and Tardos codes [45, 48] (refinement of BS codes by reducing the length of code-word). Robustness of a fingerprinting scheme is crucial against such attacks in case different copies of the dbSNP is breached at the same time or multiple SPs holding different copies of the dbSNP collude. Our proposed Gen-Scope is readily to be incorporated with the Tardos codes [48] to achieve dbSNP fingerprinting with privacy guarantee and robustness against collusion attack. In particular, Alice (the dbSNP owner) only needs to replace the fingerprint generation step (Line 2 of Algorithm 1) with the Tardos codes [48] shown as in Algorithm 3, where p is the probability of changing one bit of a SNP entry, which is also the probability of a specific element in Tardos codes taking value 1. The PDF of p is parameterized by t∈(0,0.5). As will be clear later, the value of t determines whether Gen-Scope can achieve ϵ-LDP and robustness against collusion attacks at the same time.

**Algorithm 3:** Tardos code generation.

1	Sample a random variable p from probability density function $f\left(p;t\right)=\frac{1}{2\;\text{arcsin}\left(1-2t\right)}\frac{1}{\sqrt{p-\left(1-p\right)}},\;t\in \left(0,0.5\right)$.
2	Generate the Tardos fingerprint string, i.e., $\text{f}\sim \text{Bernoulli}\left(p\right)$

After generating a customized Tardos code for a specific SP, Alice can embed the code into the dbSNP by following the same procedure in the proposed algorithm (i.e., applying (2) while switching f with the Tardos code). For completeness, we summarize the steps to generate privacy-preserving fingerprinted dbSNP copies with collusion resistance in Algorithm 4.


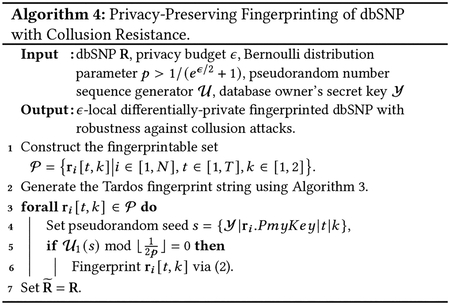


Once having spotted a pirated copy of the shared dbSNP, Alice will first use the same steps discussed in Algorithm 2 to extract the fingerprint bit-string (denoted as ${\text{f}}^{\prime }$), and then perform the accusation steps presented in [48] to hold one or more colluders (malicious SPs) responsible. To be more specific, for each SP with public ID, Alice computes the accusation sum via

(4) $$S_{ID_{\text{external}}}=\sum_{l}^{\left|{\text{f}}^{\prime }\right|}{\text{f}}_{l}^{\prime }U\left({\text{f}}_{l},p\right),\;U\left({\text{f}}_{l},p\right)=\left\{\begin{array}{ll} \sqrt{\frac{1-p}{p}} & {\text{f}}_{l}=1\\ -\sqrt{\frac{p}{1-p}} & {\text{f}}_{l}=0 \end{array}\right.,$$

and finally accuses this SP as guilty if $S_{\text{ID}}>Z$ (Z is a predetermined accusation threshold).

To defend against collusion attack, the goal of the database owner is to identity at least one pirate of the guilty coalition [48]. Thus, the robustness against collusion attack is usually quantified using the desired probability of an innocent SP gets falsely accused, i.e., $\beta_{1}$, and the probability of failing to accuse any of the colluders (malicious SPs), i.e., $\beta_{2}$. Before establishing the privacy guarantee using the Tardos codes, we first review its original robustness guarantee as follows, which is a restatement of Corollary 1 in [46].

Theorem 3 (Robustness of Tardos Codes [46]). *Given at most* $c_{0}$ *colluders* $\left(c_{0}\geq 2\right),\;c_{0}t<1,\;t\in (0,0.5)$, $0<\beta_{1}\ll \beta_{2}\ll 1$. *If the database owner uses Tardos codes with length* $L\geq 4\pi^{2}c_{0}^{2}\text{ln}\left(\beta_{1}^{-1}\right)$ *and accusation threshold* $Z=20c_{0}\left\lceil \text{ln}\left(\beta_{1}^{-1}\right)\right\rceil$, *then the probability of an innocent SP being falsely accused is at most* $\beta_{1}$, *and the probability of failing to accuse any of the colluders is at most* $\beta_{2}$.

Now, we unify ϵ-LDP guarantee and robustness against collusion attack by tuning t (parameter of the Tardos codes, Line 1 of Algorithm 3). By adapting the theoretical results established in [46] (in particular Corollary 1 in [46], which improves the original Tardos codes in [48]), we can arrive at the following conclusion.

Theorem 4. *Given at most* $c_{0}$ *colluders (*$c_{0}\geq 2),\;c_{0}t<1,\;t\in (0,0.5)$, $0<\beta_{1}\ll \beta_{2}\ll 1$ (here t *is the parameter of the probability density function used in Tardos code, and $\beta_{1}$ and* $\beta_{2}$ *are probability of false accusation and miss accusation), if Gen-Scope incorporating the Tardos codes satisfy the following conditions*

1. *Tardos codes with length* $L\geq 4\pi^{2}c_{0}^{2}\text{ln}\left(\beta_{1}^{-1}\right)$ *and accusation threshold* $Z=20c_{0}\left\lceil \text{ln}\left(\beta_{1}^{-1}\right)\right\rceil$,
2. $c_{0}\leq e^{\epsilon /2}+1$ *and* $t\in \left[\frac{1}{e^{\epsilon /2}+1},\frac{1}{c_{0}}\right)$,

*then, the fingerprinted dbSNP provides the following guarantees*

- *the probability of an innocent SP being falsely accused is at most* $\beta_{1}$, *and probability of failing to accuse any of the colluders is at most* $\beta_{2}$,
- ϵ-*local differential privacy with respect to each SNP entry*.

Proof. The pair of condition (1) and guarantee (i) is achieved by directly applying the theoretical results of Tardos codes (refer the proof to Corollary 1 in [46] for details). The pair of condition (2) and guarantee (ii) holds, because one can easily verify that f(p) (step 1 of Tardos code generation) only spans the interval of [t,1−t]. It suggests $p\geq t\geq \frac{1}{e^{\epsilon /2}+1}$, which is a sufficient condition to invoke Theorem 1 and 2. □

**Figure 2:**
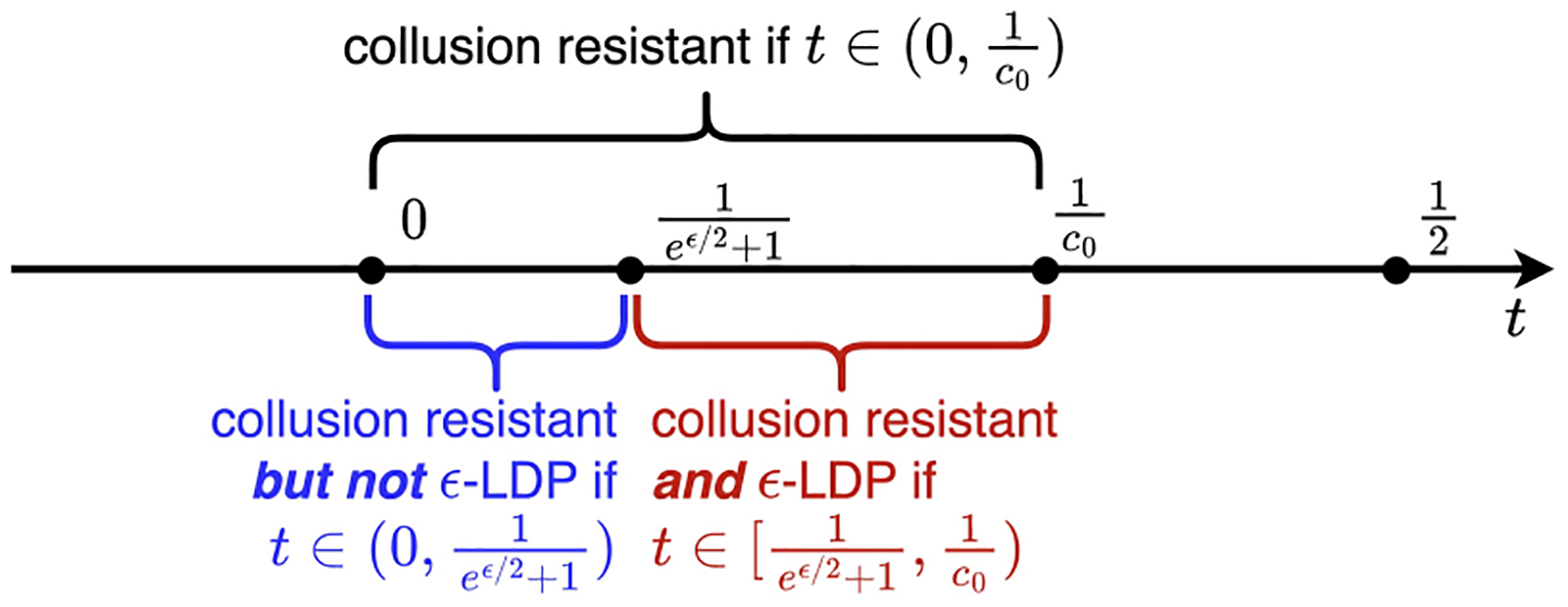
Range of t when ϵ-LDP is attained (or not) by the Tardos codes with different parameter t.

To better interpret the results of Theorem 3 and Theorem 4, we visualize the range of t where ϵ-LDP is attained or not by using the Tardos codes in Figure 2. To be more specific, the fingerprinted database can withstand collusion attack of at most $c_{0}$ colluders as long as $t\in \left(0,\frac{1}{c_{0}}\right)$. Moreover, if the Tardos code parameter (i.e., t) is chosen appropriately, i.e., $t\in [\frac{1}{e^{\epsilon /2}+1},\frac{1}{c_{0}})$, the fingerprinted database can also achieve ϵ-LDP. However, the maximum collusion size is limited to $\left\lfloor e^{\epsilon /2}+1\right\rfloor$. On the contrary, if $t\in \left(0,\frac{1}{e^{\epsilon /2}+1}\right)$, the resulting fingerprinted database cannot achieve the ϵ-LDP guarantee.

Note that another challenge in practical use of DP is the cumulative privacy loss when the same statistics are repeatedly calculated and released using the same DP mechanism. This is also true for sharing a privacy-preserving dbSNP with multiple SPs. If the dbSNP is shared to $c_{0}$ receipts, we consider the privacy leakage will compose linearly, e.g., each SNP is protected under $\epsilon c_{0}\text{-LDP}$.

## 7 QUANTIFYING PRIVACY AND COPYRIGHT ROBUSTNESS GUARANTEE

As utility is one of the most important metrics in biomedical research, we compare the accuracy of fingerprinted dbSNP and GWAS statistics achieved by Gen-Scope and the naïve two-step approach (LDP followed by fingerprinting) in Section 8. To achieve a fair comparison, we require that both Gen-Scope and the two-step approach provide an identical privacy and copyright protection guarantees.

**Same privacy guarantee.** Since both Gen-Scope and the two-step approach provide local differential privacy, to achieve the same privacy guarantee, we can set the same ϵ value for both of them.

Now, we provide a novel approach to quantify the robustness (copyright protection guarantee) against random bit flipping attack and collusion attack at the same time. The robustness against correlation attacks will be empirically evaluated in Section 8.

**Same copyright protection guarantee.** We require both Gen-Scope and the two-step approach achieve the same robustness against random bit flipping attack (with flipping probability 1/2) and collusion attack (discussed in Section 4.1.2). This is because random bit flipping attack is the most powerful among simple attacks^2^, as the flipped SNP entries may create a fingerprint pattern that misleads Alice during the fingerprint extraction phase [24, 27]. We also consider the robustness against collusion attack, because it has become increasingly common in data sharing. Since the robustness of a fingerprinting scheme is related to the percentage (density) of fingerprinted bits (denoted as γ), we need to derive a closed form expression connecting γ with robustness against random bit flipping attack and collusion attack. Note that the robustness against correlation attack depends on the specific correlation models and vary with the database [24, 27], thus, we only consider empirical study on correlation attacks followed by collusion attacks.

We first discuss the robustness against random bit flipping attack. Given a specific value of γ(γ∈(0,1)), the number of fingerprinted bits in a dbSNP is γ2NT(N and T are the number of rows and columns of dbSNP and each SNP is coded using 2 bits). Suppose the length of the fingerprint bit-string is L, then, each bit of the fingerprint bit-string is used γ2NT/L times approximately (because each fingerprint bit is randomly sampled from a length L string). Thus, in order to compromise a particular fingerprint bit, a malicious SP needs to alter it for at least τγ2NT/L times (where τ∈(0.5,1) is Alice’s confidence in recovering a fingerprint bit via majority vote in Algorithm 2). Hence, the probability (denoted as $p_{\text{rnd}}$) that a malicious SP can successfully compromise a fingerprint bit via random bit flipping attack is prnd=∑i=τγ2NT/Lγ2NT/Lγ2NT/Li12γ2NT/L.

To achieve identical robustness against random bit flipping attack, we require $p_{\text{rnd}}$ being upper bounded by a specific threshold (Ω) for both Gen-Scope and the two-stage approach. Then, the required percentage of fingerprinted bits $\left(\gamma^{*}\right)$ can be achieved via

(5) γ*=argminγ|∑i=τγ2NT/Lγ2NT/Lγ2NT/Li12γ2NT/L≤Ω.

$\gamma^{*}$ can be interpreted as the smallest percentage of fingerprinted bits that can guarantee that random bit flipping attack succeeds with probability at most Ω(Ω≪1). Clearly, it is computationally prohibitive to directly solve for $\gamma^{*}$ in (5). Here, we provide an approximate solution to (5). First, due to the symmetry of Binomial distribution, (5) is equivalent to

(6) γ*=argminγ∑i=0(1−τ)γ2NT/Lγ2NT/Li12γ2NT/L≤Ω.

In particular, (6) is the cumulative density function (CDF) of the Binomial distribution with parameter γ2NT/L (number of trials) and $\frac{1}{2}$ (probability of each trial being successful), i.e., Binomial $\left(\gamma 2NT/L,\frac{1}{2}\right)$. Such CDF is evaluated at γ2NT/L−τγ2NT/L. On the other hand, (5) is the complementary CDF (i.e., the tail distribution) of the same distribution evaluated at τγ2NT/L.

Next, we approximate Binomial $\left(\gamma 2NT/L,\frac{1}{2}\right)$ using a Gaussian distribution with mean $\gamma 2NT/L\times \frac{1}{2}$ and variance $\gamma 2NT/L\times \frac{1}{2}\times \frac{1}{2}$, i.e., 𝒩(γNT/L,γNT/2L).^3^ Then, the value of Gaussian random variable leading to cumulative probability Ω can be calculated via $X^{*}=\Phi^{-1}(\Omega )\times \sqrt{\gamma NT/2L}+\gamma NT/L$, where $\Phi^{-1}(\cdot )$ returns the inverse value of standard Gaussian CDF given a probability Ω. Then, by letting $X^{*}\geq (1-\tau )\gamma 2NT/L$, we can solve for γ as $\sqrt{\gamma }\geq \sqrt{\frac{L}{NT}}\frac{\Phi^{-1}(\Omega )}{\sqrt{2}(1-2\tau )}$, which suggests $\gamma^{*}=\frac{L}{2NT}{\left(\frac{\Phi^{-1}(\Omega )}{1-2\tau }\right)}^{2}$.

Based on Theorem 4, given a predetermined probability $\beta_{1}$, we can achieve robustness against collusion attack involving at most $c_{0}$ colluders as long as the length of the fingerprint bit-sting satisfies $L\geq 4\pi^{2}c_{0}^{2}\text{ln}\left(\beta_{1}^{-1}\right)$. Hence, to simultaneously achieve robustness (copyright guarantee) against random bit flipping attack and collusion attack, we require the percentage of fingerprinted bits satisfy

(7) $$\gamma \geq \left(\frac{4\pi^{2}c_{0}^{2}\text{ln}\left(\beta_{1}^{-1}\right)}{2NT}\right)\times {\left(\frac{\Phi^{-1}\left(\Omega \right)}{1-2\tau }\right)}^{2}.$$

Then, we obtain the following claims about the privacy and copyright guarantees of Gen-Scope and the two-stage approach.

Claim 1. *For any* ϵ>0, *Gen-Scope achieves* ϵ-LDP, *robustness against random bit flipping attack (with failure probability at most* Ω), *and robustness against collusion attack with* $c_{0}$ *colluders (with false accusation probability at most $\beta_{1}$) if* $\frac{2}{e^{\epsilon /2}+1}\geq \gamma$. *To this end, Gen-Scope will change* $1-{\left(\frac{e^{\epsilon /2}}{e^{\epsilon /2}+1}\right)}^{2}$ *of the SNP entries*.

Proof. According to Theorem 1, Gen-Scope achieves ϵ-LDP if the probability of a bit of SNP is changed due to fingerprint insertion satisfies $p\geq \frac{1}{e^{\epsilon /2}+1}$, i.e., the probability of a bit of SNP is xored by 1 is larger than $\frac{1}{e^{\epsilon /2}+1}$. Since there is equal probability that a bit of SNP is not changed due to fingerprint insertion (i.e., a bit of SNP is xored by 0), it implies the percentage of fingerprinted bits (a bit of SNP being either xored by 1 or 0) caused by Gen-Scope is at least $2p=\frac{2}{e^{\epsilon /2}+1}$. To satisfy the required robustness against random flipping attack and collusion attack, it is sufficient to make $\frac{2}{e^{\epsilon /2}+1}\geq \gamma$ (where γ is provided in (7)). Since Gen-Scope changes each SNP bit independently with probability p, the percentage of changed SNP entries in Gen-Scope is $1-(1-p{)}^{2}$. By plugging in $p=\frac{1}{e^{\epsilon /2}+1}$, we obtain the conclusion. □

Claim 2. *For any* ϵ>0, *the two-stage approach (LDP followed by fingerprinting) achieves* ϵ-LDP, *robustness against random bit flipping attack (with failure probability at most* Ω*), and robustness against collusion attack with* $c_{0}$ *colluders (with falsely accusation probability at most* $\beta_{1}$*) if it first changes the value of each SNP with probability* $\frac{1}{e^{\epsilon }+2}$ *and then fingerprints at least* γ *bits of the new SNPs, where* γ *is given in (*7*). To this end, the two-stage approach will change approximately* $\frac{1}{e^{\epsilon }+2}+1-{\left(1-\frac{\gamma }{2}\right)}^{2}$ *of the SNP entries*.

Proof. In the first step of the two-stage approach, to achieve ϵ-LDP on SNP data, a random response scheme is applied [52], which shares an incorrect value of a specific SNP with probability $\frac{1}{e^{\epsilon }+m-1}$, where m=3 is the number of possible values a SNP can take. Then, to further make the perturbed dbSNP satisfy the required robustness against random flipping attack and collusion attack, the two-stage approach needs to change at least γ bits of the SNPs. Since during fingerprinting insertion each selected bit will be xored by 1 or 0 with equal probability, the fingerprinting stage will change a bit of a SNP with probability $\frac{\gamma }{2}$, which leads to $1-{\left(1-\frac{\gamma }{2}\right)}^{2}$ changed SNP entries. □

## 8 EXPERIMENT RESULTS

We evaluate the developed Gen-Scope using a real world large-sclae dbSNP (i.e., the HapMap dataset [16, 22]), which is a collection of SNP sequences of 15,000 individuals. Each individual has 156 SNPs.

### 8.1 Ethical Considerations

Our research does not entail direct engagement with human participants, thereby minimizing ethical risks commonly associated with genomic data collection. The HapMap genomic dataset used in this study is a public dataset and its participants’ genomic data is collected with informed consent, privacy protection, transparency, and integrity [49].

The primary concern of using this HapMap dataset and genomic dataset in general is that the experiments may reveal information about individuals’ health risks, ancestry, or other sensitive traits, which could have significant implications for their well-being and rights. Our proposed Gen-Scope precisely addresses this concern by making sure that all experiment results are protected under local differential privacy.

When utilizing genomic datasets, data curators, researchers, and service providers are obligated to uphold the trust of participants and possess mechanisms to trace the origins of data breaches. Our proposed Gen-Scope also addresses this concern by incorporating imperceptible fingerprints, preventing potential data leakage and facilitating the tracing of data provenance.

### 8.2 Comparison with the Two-Stage Approach

First, we compare Gen-Scope with the two-stage approach by evaluating the accuracy and GWAS statistics of the fingerprinted dbSNPs when they provide the same privacy and copyright guarantees.

#### 8.2.1 Comparing accuracy of dbSNPs.

According to (7), the copyright guarantee of a fingerprinted dbSNP is determined by 4 parameters, i.e., (i) Ω: the probability upper bound that random bit flipping attack can successfully compromise a fingerprint bit, (ii) τ: Alice’s confidence when recovering a specific fingerprint bit in fingerprint extraction phase (Algorithm 2), (iii) $\beta_{1}$: the probability of false accusation in collusion attack, and (iv) $c_{0}$: the number of colluders. We investigate the impact of each parameter while keeping the others fixed. Particularly, for each obtained γ, we first achieve ϵ-LDP guarantee for Gen-Scope by solving $\frac{2}{e^{\epsilon /2}+1}=\gamma$ (Claim 1), i.e., $\epsilon =2\;\text{ln}\left(\frac{2}{\gamma }-1\right)$. Next, we generate Tardos codes that satisfy the two conditions in Theorem 4, and finally insert Tardos code into a dbSNP by applying Algorithm 4. The obtained fingerprinted dbSNP will satisfy ϵ-LDP and copyright guarantee (with provided $\Omega ,\;\tau ,\;\beta_{1}$, and $c_{0}$). Then, to let the two-stage approach achieve the same LDP and copyright guarantee, we replace each SNP value with an incorrect value with probability $\frac{1}{e^{\epsilon }+2}$, where $\epsilon =2\;\text{ln}\left(\frac{2}{\gamma }-1\right)$, and then apply a previously developed genomic database fingerprinting scheme to mark γ of the bits in the perturbed dbSNP (i.e., run Algorithm 1 in [24] with $\gamma =\gamma_{r}\gamma_{l}$, where $\gamma_{r}$ (or $\gamma_{l}$) is the row-(or column-)wise fingerprint density).

**Figure 3:**
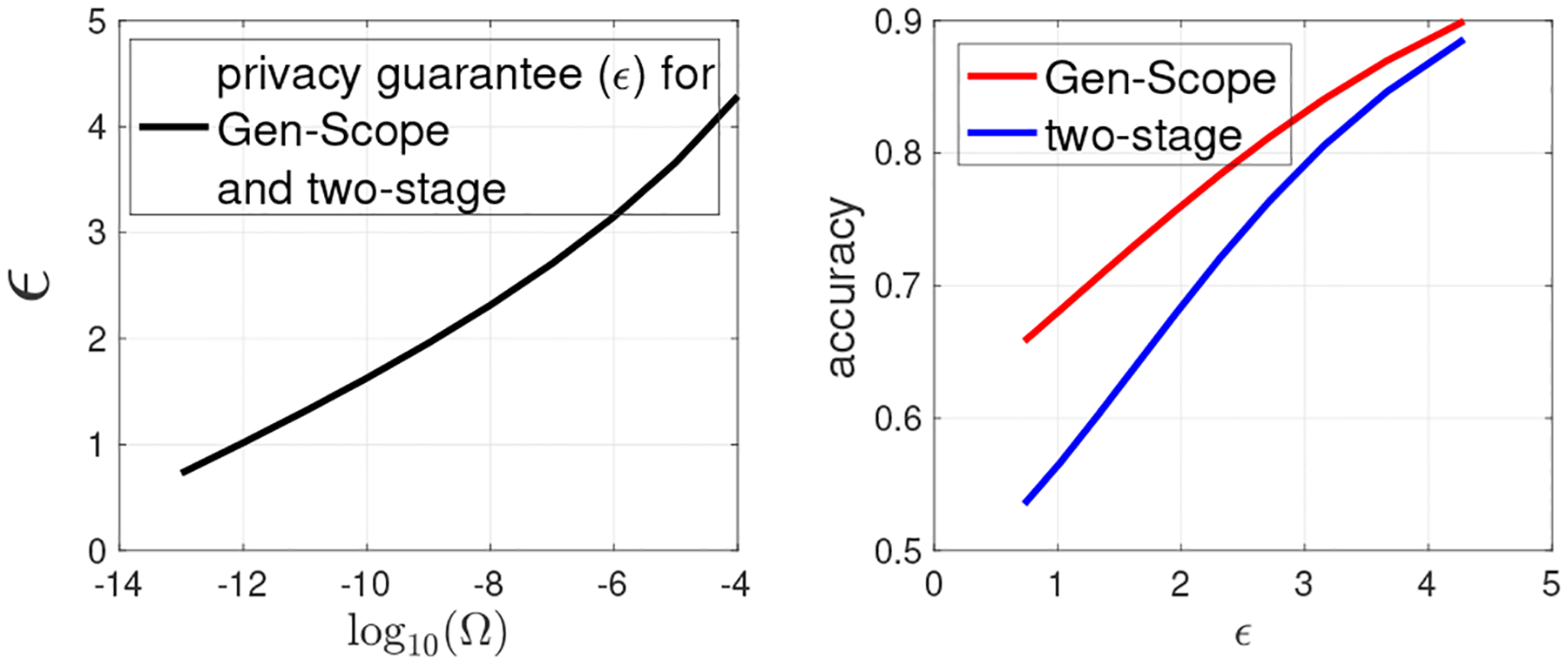
(1) Gen-Scope versus the two-stage approach under the same privacy and copyright guarantees. Fixing τ=0.7, $\beta_{1}=10^{-5},\;c_{0}=5$, varying Ω.

**Figure 4:**
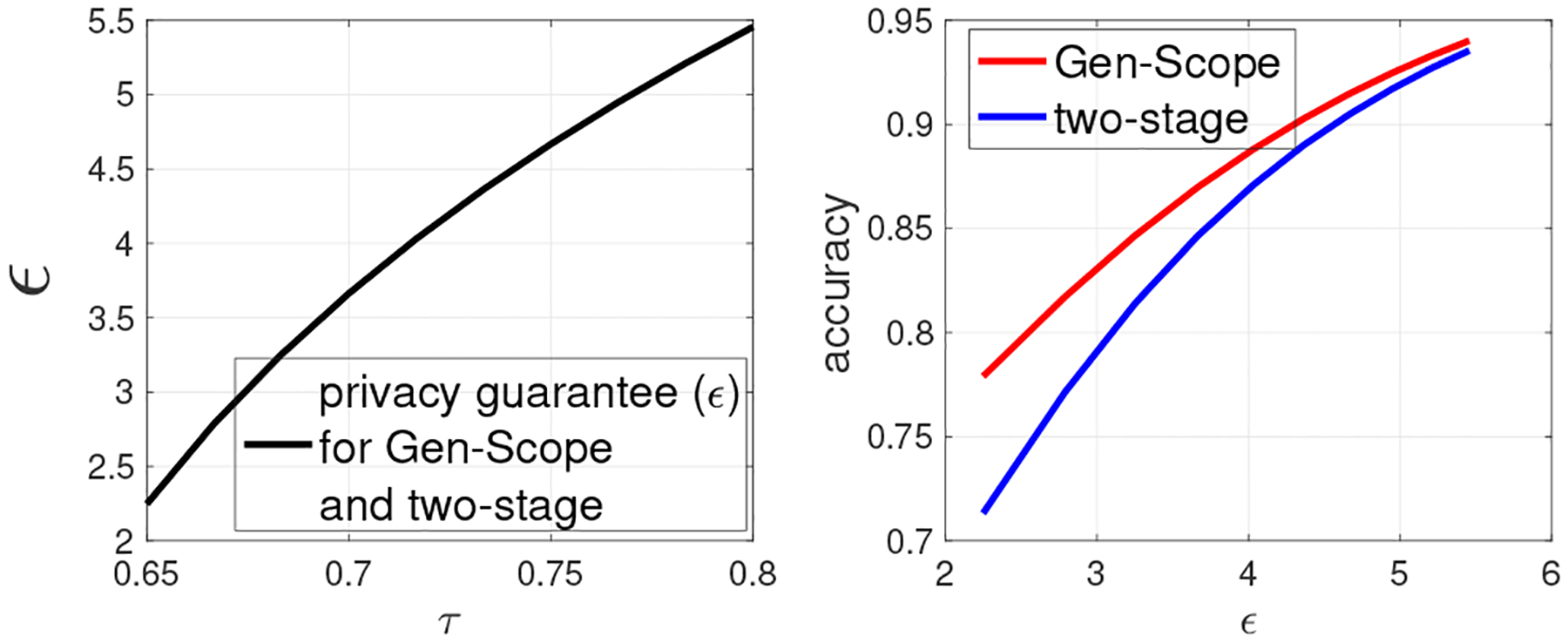
(2) Gen-Scope versus the two-stage approach under the same privacy and copyright guarantees. Fixing $\Omega =\beta_{1}=10^{-5},\;c_{0}=5$, varying τ.

**Figure 5:**
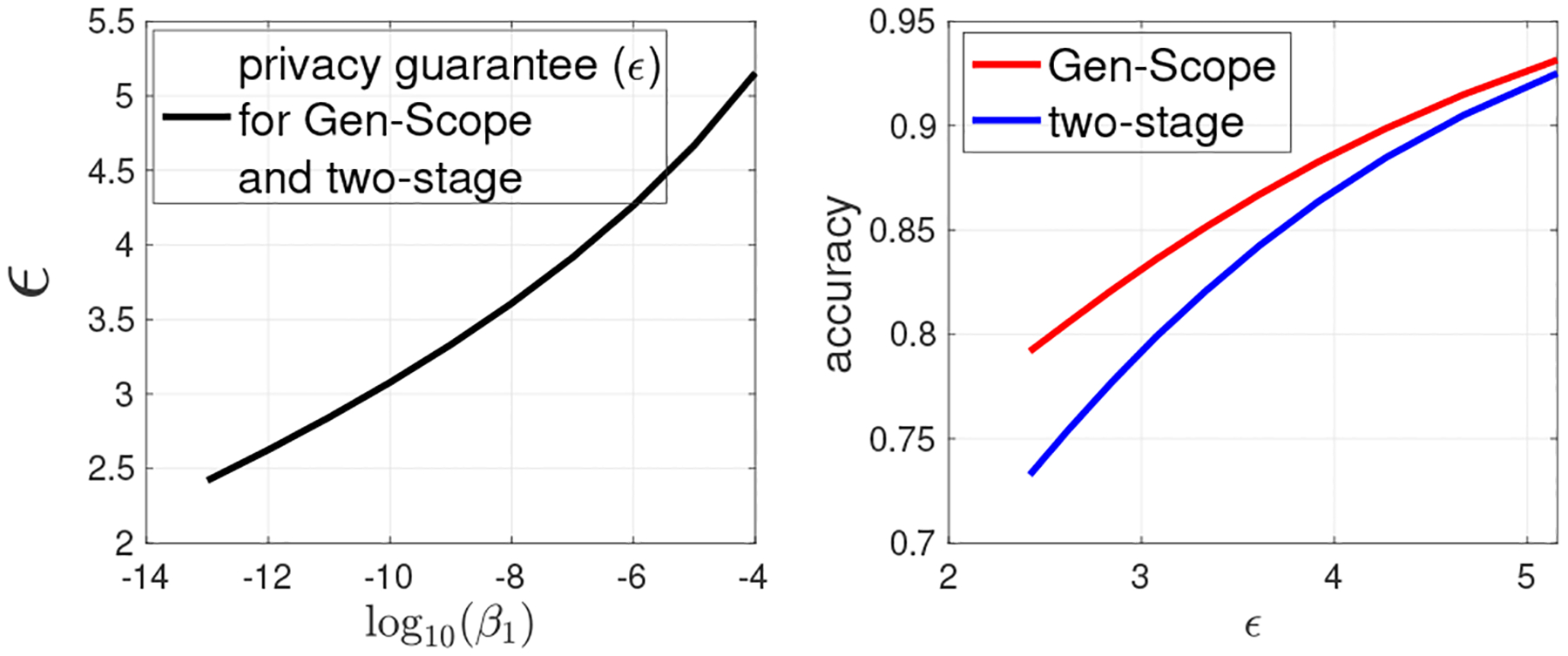
(3) Gen-Scope versus the two-stage approach under the same privacy and copyright guarantees. Fixing $\tau =0.75,\;\Omega =10^{-5},\;c_{0}=5$, varying $\beta_{1}$.

**Figure 6:**
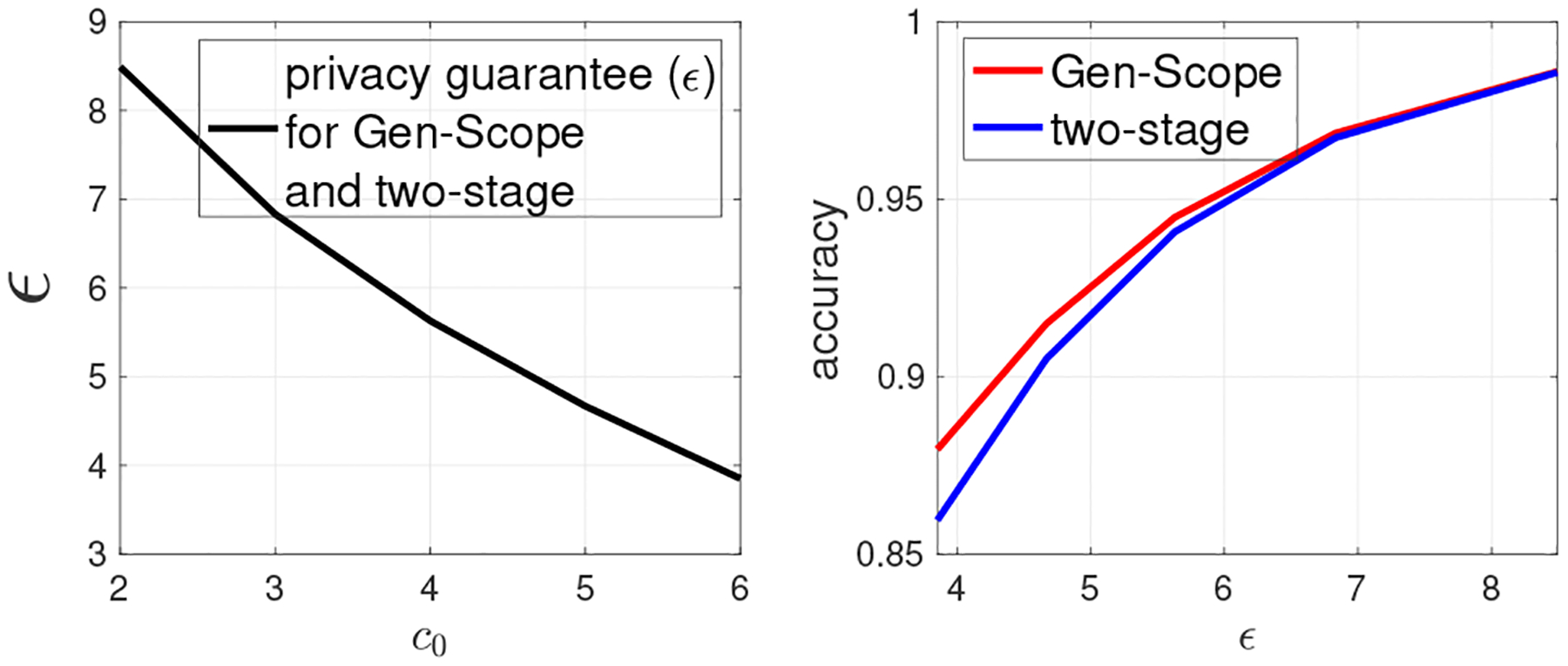
(4) Gen-Scope versus the two-stage approach under the same privacy and copyright guarantees. Fixing $\Omega =0.75\;\Omega =\beta_{1}=10^{-5}$, varying $c_{0}$.

In Figure 3–6, we obtain the various privacy guarantees (ϵ) by varying $\Omega ,\;\tau ,\;\beta_{1}$, and $c_{0}$, respectively, and also compare the accuracy of Gen-Scope and the two-stage approach using the obtained ϵ. Specifically, in Figure 3, we fix $\tau =0.7,\;\beta_{1}=10^{-5},\;c_{0}=5$, and vary Ω in [10^−13^, 10^−4^]. On the left panel of Figure 3, we plot the LDP guarantee (ϵ) versus ${\text{log}}_{10}(\Omega )$. We see that privacy guarantee becomes weaker (ϵ increases) as Ω increases. This is because the larger the value of Ω, the less the robustness becomes against random flipping attack, which implies that the inserted fingerprint is sparse, i.e., ϵ has a larger value. On the right panel of Figure 3, given the obtained ϵ, we plot the accuracy of fingerprinted dbSNPs obtained by both approaches when Ω increases. Clearly, Gen-Scope always achieves higher accuracy than the two-stage approach, because it unifies privacy preservation and copyright protection into one step. For both approaches, accuracy increases with Ω, as higher Ω implies lower fingerprinted bits, i.e., smaller value of γ (see (7)).

In Figure 4, we fix $\Omega =\beta_{1}=10^{-5},\;c_{0}=5$, and vary τ in [0.65, 0.8] (Alice’s confidence in majority voting in Algorithm 2). The left panel of Figure 4 shows that privacy guarantee becomes weaker when τ increases. Since Ω quantifies the probability that random bit flipping attack successfully compromises τ of those dbSNP bits that are marked by a specific fingerprint bit, if τ increases for a fixed Ω, it implies that fingerprinting robustness decreases, which suggests weaker privacy. From the right panel of Figure 4, we observe as τ increases (i.e., ϵ increases), Gen-Scope also outperforms the two-stage approach.

In Figure 5, we fix $\tau =0.75,\;\Omega =10^{-5},\;c_{0}=5$, and vary $\beta_{1}$ in [10^−13^, 10^−4^]. The left panel of Figure 5 shows that privacy guarantee becomes weaker when $\beta_{1}$ increases. This is due to the reason that the higher value of $\beta_{1}$ implies less fingerprinting robustness against collusion attack, which further suggests weaker privacy. From the right panel of Figure 5, we observe that Gen-Scope still outperforms the two-stage approach in terms of the accuracy of obtained dbSNP.

In Figure 6, we fix $\tau =0.75,\;\Omega =\beta_{1}=10^{-5}$, and vary $c_{0}$ from 2 to 6. From the left panel of Figure 6, we can see privacy becomes stronger (i.e., ϵ decreases) as $c_{0}$ increases. This is because higher value of $c_{0}$ means that the fingerprinted dbSNP is robust against collusion attack involving more colluders, which leads to a higher value of γ and requires more bits to be fingerprinted. Thus, this also leads to decreasing accuracy of fingerprinted dbSNP obtained by the two approaches as shown in the right panel of Figure 6. However, Gen-Scope still achieves higher accuracy, because it attains the required privacy preservation and copyright protection guarantee via one-shot noise injection.

From Figure 3–6, we observe that the privacy guarantee and fingerprinting robustness is limited under high ϵ regime. In particular, when ϵ>4, the proposed Gen-Scope method leads to similar utility with the two-stage approach, and the shared genomic databases obtained using both methods will have poor privacy guarantees and fingerprinting robustness. Thus, to fulfill the three requirements (security, privacy, utility discussed in Section 4.1.1) when sharing genomic database, the database owner need to choose an appropriate ϵ. For the database considered in this work, when ϵ is approximately 3, the proposed Gen-Scope has clear advantage over the two-stage approach in terms of all fingerprint robustness, privacy, and GWAS utility. We defer the task of determining the optimal ϵ that achieves a suitable balance between utility, privacy, and robustness to future research.

#### 8.2.2 Comparing GWAS statistics.

To evaluate the utility of GWAS statistics, we investigate the consistency of SNP-phenotype association of dbSNPs obtained using various methods and compare them with the SNP-phenotype association obtained from the original dbSNP. In particular, we first obtain the set of top-K percentage of SNPs having strong associations with a phenotype (i.e., top-K percentage SNPs with the lowest p-values) from the original dbSNP and denote this set as the ground-truth set. Next, we get the new sets of top-K SNPs from (i) Gen-Scope, (ii) two-stage approach (i.e., LDP followed by fingerprinting in [24]), (iii) only LDP perturbation of the original dbSNP (i.e., no copyright protection is attained), and (iv) only fingerprinting the original dbSNP (i.e., no privacy guarantee is attained). Finally, we evaluate the consistency by counting the fraction of overlapping between each of the new sets and the ground-truth set.

**Figure 7:**
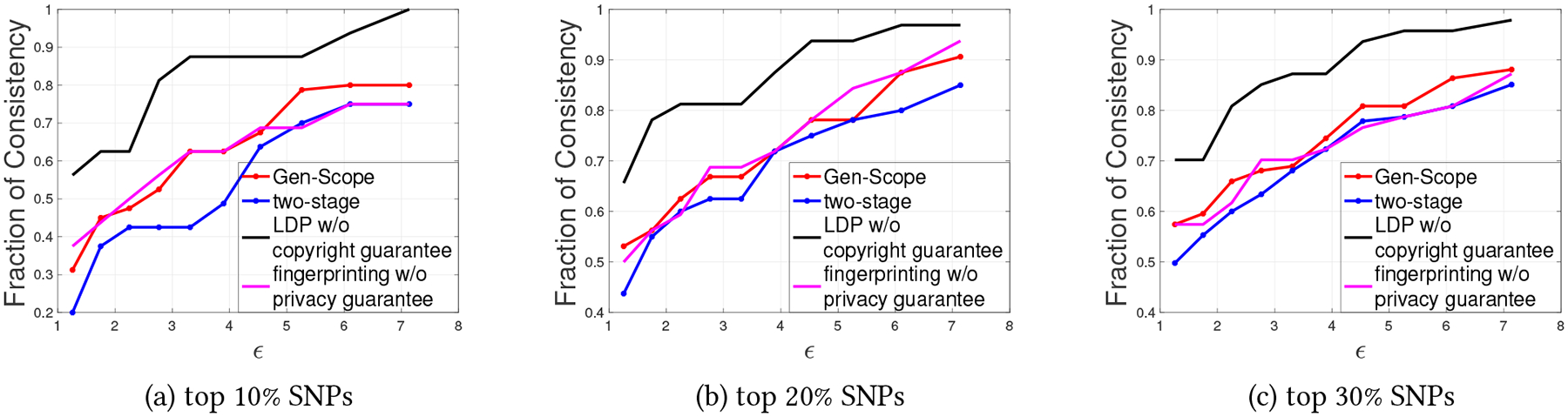
Comparison of the consistency of SNP–phenotype association achieved by Gen-Scope, the two-stage approach, LDP (without copyright guarantee), and fingerprinting in [24] (without privacy guarantee).

In this experiment, we set $\tau =0.85,\;\Omega =\beta_{1}\in \left\{10^{-13},\cdots ,10^{-4}\right\}$, and obtain γ using (7) and set $\epsilon =2\;\text{ln}\left(\frac{2}{\gamma }-1\right)$. In Figure 7, we plot the fraction of consistent SNP-phenotype association when K is 10,20, and 30. Clearly, Gen-Scope can always achieve higher consistency frequency than the two-stage approach. For example, when we consider the top-10% of the SNPs having strong associations with a phenotype, Gen-Scope can preserve about more than 20% of those SNPs compared with the two-stage approach when ϵ is about 3.3.

**Figure 8:**
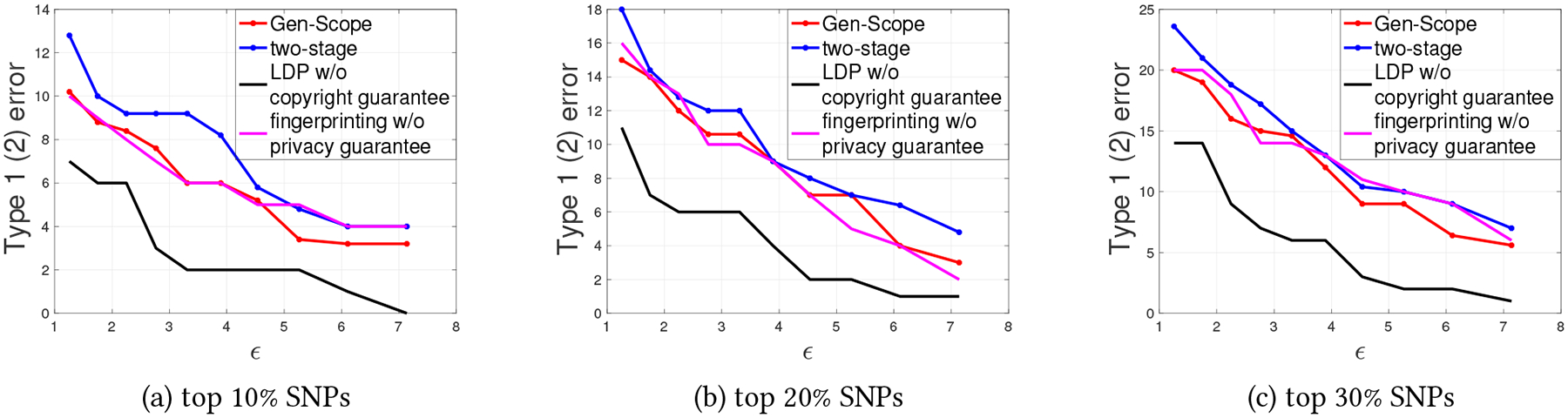
Comparison of the Type 1 (or Type 2) error of the SNP–phenotype association achieved by Gen-Scope, the two-stage approach, LDP (without copyright guarantee), and fingerprinting in [24] (without privacy guarantee).

Additionally, we also present the Type 1 error (known as the false positives). It is the number of the SNPs erroneously identified as having strong associations by various mechanisms, when in reality, they have weak associations according to the ground-truth set. Specifically, these SNPs do not belong to the top-K percentage of SNPs with the lowest p-values. Note that for any fixed K, the number of strongly and weakly associated SNPs are also fixed. Thus, a false positive SNP must corresponds to a false negative SNP (the SNP erroneously identified as having weakly associated). As a result, Type 1 error equals to Type 2 error in our study. We show the experiment results in Figure 8 when K is 10,20, and 30. Clearly, Gen-Scope can always achieve lower Type 1 or (Type 2) error than the two-stage approach as it modifies less SNPs to achieve both privacy guarantee and fingerprinting robustness.

### 8.3 Robustness against Random Bit Flipping Attack and Collusion Attack

**Figure 9:**
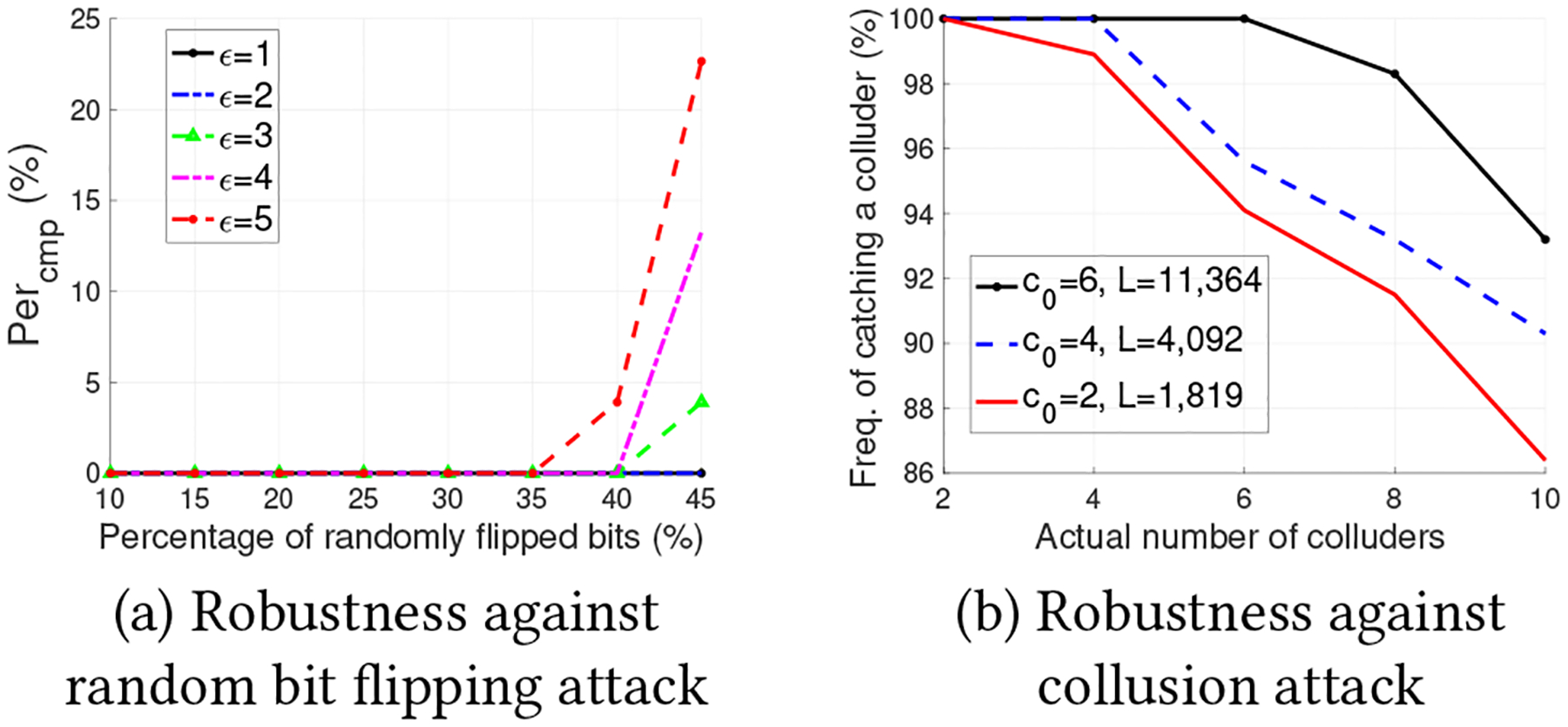
Investigation of robustness of Gen-Scope against random bit flipping attack and collusion attack. (a): Robustness against random bit flipping when ϵ∈{1,2,3,4,5} and the percentage of random flipped bits increases from 10% to 45%. (b) Robustness against against Collusion attack consider 2, 4, or 6 colluders, i.e., $c_{0}\in \{2,\;4,\;6\}$, when there are actually $c_{1}$ colluders, and $c_{1}\in \{2,3,\cdots ,10\}$.

Next, to verify the fingerprinting robustness of Gen-Scope, we launch random bit flipping attack and collusion attack on the obtained fingerprinted dbSNP. In particular, we let a malicious SP randomly flip a certain percentage of the bits in its received copy of dbSNP, and then extract the fingerprint bit-string from the compromised dbSNP, compare the extracted string with the original fingerprint bit-string that is generated for this SP, and finally compute the percentage of compromised fingerprint bits (${\text{Per}}_{\text{cmp}}$ in Section 4.2). In Figure 9(a), by selecting the privacy budget ϵ from {1, 2, 3, 4, 5}, we plot ${\text{Per}}_{\text{cmp}}$ when the percentage of flipped bits increases from 10% to 45%. Clearly, even with the least guarantee on privacy and copyright protection (i.e., ϵ=5), the malicious SP can only compromise less than 23% of the fingerprint bits even though it sacrifices the utility of the dbSNP by flipping 45% of the bits. This suggests a very high robustness against random bit flipping attack, because as long as less than half of the fingerprint bits are compromised, Alice is able hold the malicious SP responsible for the data leakage [24, 27].

To test the robustness against collusion attack, we fix $\beta_{1}=10^{-5}$ (see Theorem 4) and let Alice generate Tardos code by only considering 2, 4, or 6 colluders, i.e., $c_{0}\in \{2,4,6\}$, when there are actually $c_{1}$ colluders, and $c_{1}\in \{2,3,\cdots ,10\}$. We let the the coalition employ the majority strategy [9, 32], where colluding SPs compare their received dbSNPs and replace each bit with the majority of that bit in all the copies. Then, after extracting the fingerprint from the pirated dbSNP, we calculate the frequency of detecting at least one of the $c_{1}$ colluders. The frequency is obtained by repeating the experiment 30 times. We plot the results in Figure 9(b). We observe that as long as $c_{0}\leq c_{1}$, Alice can always trace one of the colluders. Even when $c_{1}>c_{0}$, the successful tracing frequency is still high, e.g., when there are 10 colluders actually, but Alice only consider $c_{0}=4$, she is still able to accuse one of the 10 colluders with chance larger than 90%. This suggests that the proposed Gen-Scope is also robust against the collusion attack.

### 8.4 Robustness against Correlation Attacks Followed by Collusion Attack

Now we empirically investigate the robustness of the proposed Gen-Scope against the most powerful attack combination, i.e., each malicious receipts independently perform correlation attacks on their own received fingerprinted dbSNP, and then forge a single copy via collusion.

Since the added privacy-preserving fingerprint changes entries in the original dbSNP, which will lead to the change of statistical relationships among genome data at different locations, the malicious SP can leverage the auxiliary correlation models (which are usually publicly available) to more accurately infer the potentially fingerprinted SNPs, and then distort the fingerprint. In this work, we consider the recently proposed correlation attacks in [24], where a malicious SP utilizes the inherent linkage disequilibrium (i.e., the joint distributions) among SNPs to identify the fingerprinted positions in a genomic database.

In favor of the attackers, we assume that the malicious SP has prior knowledge about the linkage disequilibrium (i.e., the joint distributions among each pair of the SNPs) that are directly calculated from the original dbSNP. Note that this is the most powerful correlation attack that could be launched against a given fingerprinted relational database [25]. We denote the knowledge set of joint distributions on the original dbSNP as $𝒥=\left\{J_{p,q}|p,q\in ℱ,p\neq q\right\}$, where p and q are the SNP sequences for all individuals in R at location p and q. Once receiving a fingerprinted dbSNP $\tilde{\text{R}}$, the malicious SP first calculates a new set of joint probability distributions (denoted as $\tilde{𝒥}$) based on $\tilde{\text{R}}$. Then, it compares $\tilde{𝒥}$ with its prior knowledge 𝒥, and flips the entries in $\tilde{\text{R}}$ that leads to large discrepancy between $\tilde{𝒥}$ and 𝒥. Please refer to [24, 25] for the detailed correlation attacks.

**Scenario 1.** We first release the entire dbSNP (all 156 SNPs of 15,000 individuals), and let $c_{0}=c_{1}=3$ (i.e., the Tardos code is generated by considering 3 colluders and the actual number of colluders is also 3), Alice’s confidence in recovering the fingerprint bits be 98%, random bit flipping attack can success with probability $\Omega =10^{-8}$, and false accusation happens with probability $\beta_{1}=10^{-8}$. Under this setup, Gen-Scope changes about 2.4% of the entries in the original dbSNP. After letting each 3 malicious SPs perform correlation attacks independently and merge their compromised copies by majority voting, it is interesting to find that proposed Gen-Scope is still robust, i.e., 2 out of 3 colluders can still be identified, and the accusation score $S_{ID_{\text{external}}}$ (defined in (4)) calculated for each SP is intact with or without the correlation attacks. This is because in this scenario, only 2.4% of the SNPs are modified by the Tardo code, and there are 156 columns in the dbSNP, thus the impact to the joint distributions among SNPs is negligible (i.e., the discrepancy between 𝒥 and $\tilde{𝒥}$ is small). Thus, the correlation attacks can hardly infer enough fingerprinted entries. In fact, according to the experiments in [24], it requires about 10% modifications in dbSNP to make the correlation attack successful.

**Figure 10:**
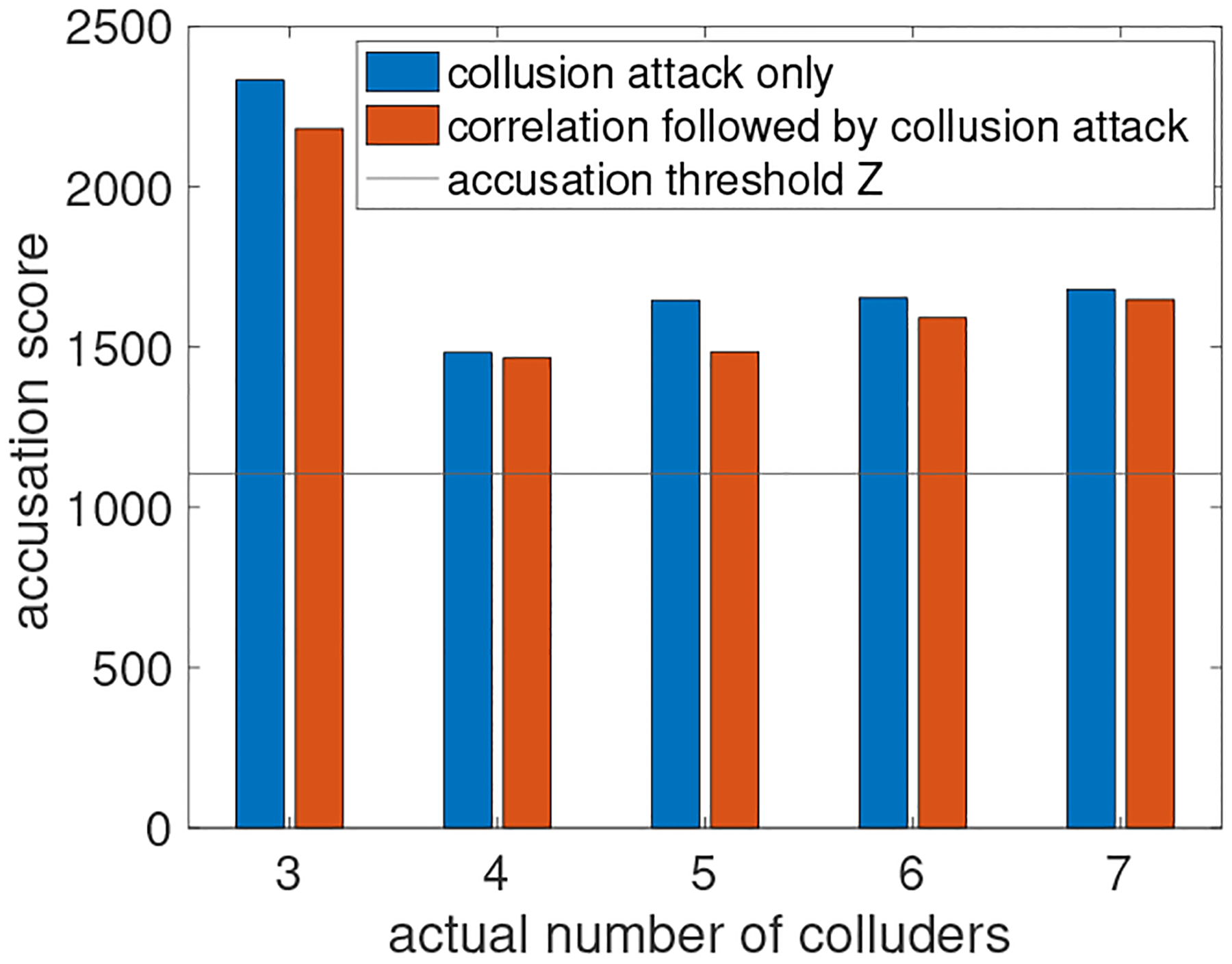
Accusation scores for the identified colluder with and without correaltion attack.

**Scenario 2.** To increase the discrepancy between 𝒥 and $\tilde{𝒥}$, we now consider releasing the first 30 SNPs of all individuals. By keeping the same parameter setups with scenario 1, the Tardos code can change about 12% of the entries in each shared copy of the dbSNP. Since there are only 30 columns, the impact to the joint distributions among SNPs will be high. We find that the proposed Gen-Scope is still robust against correlation attacks followed by collusion attacks, even if the actual number of colluders is lager than $c_{0}$. In Figure 10 we show the accusation score for one of the colluder identified by Alice when $c_{0}=3$ and there are actual $c_{1}\in \{3,4,5,6,7\}$ colluders. Clearly, the correlation attack can decrease the accusation scores (correlation attack only, blue bars) by some extent, yet, the new accusation scores (correlation attack followed by collusion, red bars) are still higher than the accusation threshold $Z=20c_{0}\left\lceil \text{ln}\left(\beta_{1}^{-1}\right)\right\rceil$. Thus, Gen-Scope is also robust against the strong combination of correlation followed by collusion attack.

## 9 CONCLUSION

In this paper, we have proposed Gen-Scope, which is the first genomic database fingerprinting scheme that can simultaneously achieve copyright protection, privacy preservation, and accurate value (utility) when sharing genomic databases. Gen-Scope attains LDP by leveraging the intrinsic randomness during fingerprint insertion. We also discussed how to improve Gen-Scope to defend against collusion attacks. We have theoretically showed that to achieve the identical privacy and copyright guarantee, Gen-Scope will change less SNPs than the two-stage approach (LDP followed by fingerprinting). The proposed Gen-Scope is also robust against correlation attacks. Experiments on a real world genomic database corroborated our theoretical findings, e.g., Gen-Scope can achieve GWAS statistics that is about 20% more accurate than the two-stage approach.

## Figures and Tables

**Table 1:** Comparison of various schemes. ✔ indicates the scheme has a certain property, and ✘ indicates the opposite.

Properties	VLDBJ’03 [2]	TDSC’05 [32]	RAID’19 [5]	Bioinformatics’21 [37]	RAID’21 [27]	ISMB’21 [24]	NDSS’23 [23]	this paper
Distinguish malicious SPs	✘	✔	✔	✘	✔	✔	✔	✔
Privacy guarantee	✘	✘	✘	✔	✘	✘	✔	✔
Collusion-attack resistant	✘	✔	✔	✘	✘	✘	✘	✔
Correlation-attack resistant	✘	✘	✘	✘	✔	✔	✘	✔
Handle relational databases	✔	✔	✘	✘	✔	✔	✔	✔

## Footnotes

1 To be more specific, genetic profiling analyzes specific regions of an individual’s SNPs and creates a unique genetic profile for that individual. By comparing the profile to a database of known profiles, it is possible to discover the individual’s identity or determine his/her relationship to other individuals in the database

2 Simple attacks are those that do not require additional information, e.g., data correlation or fingerprinted dbSNPs received by others. Examples of simple attacks include random bit flipping attack, subset (superset) attack, rounding attack [2].

3 𝒩(np,np(1−p)) is a good approximation of Binomial (n,p) when both np and n(1−p) is greater than 5 [34], which is the case for our considered experiments on dbSNP in Section 8.

## A OMITTED PROOFS

### A.1 Proof of Theorem 1

Proof. Let ${\text{r}}_{i}[t]$ and ${\text{r}}_{i}^{\prime }[t]$ be two possible values that the tth SNP of the ith individual can take in a dbSNP, i.e., ${\text{r}}_{i}[t]$, ${\text{r}}_{i}^{\prime }[t]\in \{0,1,2\}$ and ${\text{r}}_{i}[t]\neq {\text{r}}_{i}^{\prime }[t]$. Denote the last bit of ${\text{r}}_{i}[t]$ as ${\text{r}}_{i}[t,1]$ and the second to the last bit of ${\text{r}}_{i}[t]$ as ${\text{r}}_{i}[t,2]$, and suppose after fingerprinting both ${\text{r}}_{i}[t]$ and ${\text{r}}_{i}^{\prime }[t]$ become $\tilde{{\text{r}}_{i}}[t]$. Moreover, let $B_{i,t,k}$ (or $B_{i,t,k}^{\prime }$) denotes the Bernoulli random variable that is used to mark ${\text{r}}_{i}[t,k]$ or (${\text{r}}_{i}^{\prime }[t,k]$). Then, we have

$$\frac{\text{Pr}\left(ℳ\left({\text{r}}_{i}[t]\right)=\tilde{{\text{r}}_{i}}[t]\right)}{\text{Pr}\left(ℳ\left({\text{r}}_{i}^{\prime }[t]\right)=\tilde{{\text{r}}_{i}}[t]\right)}\overset{\left(a\right)}{=}\prod_{k\in \{1,2\}}\frac{\text{Pr}\left({\text{r}}_{i}[t,k]⊕B_{i,t,k}=\tilde{{\text{r}}_{i}}[t,k]\right)}{\text{Pr}\left({\text{r}}_{i}^{\prime }[t,k]⊕B_{i,t,k}^{\prime }=\tilde{{\text{r}}_{i}}[t,k]\right)}\;=\prod_{k\in \{1,2\}}\frac{\text{Pr}\left(B_{i,t,k}={\text{r}}_{i}[t,k]⊕\tilde{{\text{r}}_{i}}[t,k]\right)}{\text{Pr}\left(B_{i,t,k}^{\prime }={\text{r}}_{i}^{\prime }[t,k]⊕\tilde{{\text{r}}_{i}}[t,k]\right)}\;=\prod_{k\in \{1,2\}}\frac{p^{\left({\text{r}}_{i}[t,k]⊕\tilde{{\text{r}}_{i}}[t,k]\right)}{\left(1-p\right)}^{\left(1-{\text{r}}_{i}[t,k]⊕\tilde{{\text{r}}_{i}}[t,k]\right)}}{p^{\left({\text{r}}_{i}^{\prime }[t,k]⊕\tilde{{\text{r}}_{i}}[t,k]\right)}{\left(1-p\right)}^{\left(1-{\text{r}}_{i}^{\prime }[t,k]⊕\tilde{{\text{r}}_{i}}[t,k]\right)}}\;\overset{\left(b\right)}{=}\prod_{k\in \{1,2\}}{\left(\frac{1-p}{p}\right)}^{\left(\left({\text{r}}_{i}[t,k]-{\text{r}}_{i}^{\prime }[t,k]\right)\left(2\tilde{{\text{r}}_{i}}[t,k]-1\right)\right)}\;\leq {\left(\frac{1-p}{p}\right)}^{2},$$

where (a) is because each bit of ${\text{r}}_{i}[t]$ (or ${\text{r}}_{i}^{\prime }[t]$) is marked independently, and (b) is obtained by applying u⊕v=(1−u)v+u(1−v) for any binary u and v. Then, by making ${\left(\frac{1-p}{p}\right)}^{2}\leq e^{\epsilon }$, we complete the proof. □

### A.2 Proof of Theorem 2

Proof. Since the value of ${𝒰}_{j}(s)$ (the jth random value generated by 𝒰) is uniformly distributed for a given s [10], we have $\text{Pr}\left({𝒰}_{1}(s)\;\text{mod}\;\left\lfloor \frac{1}{2p}\right\rfloor =0\right)=1/\left\lfloor \frac{1}{2p}\right\rfloor >2p$. Similarly, $\text{Pr}(x=0)=\frac{1}{2}$, thus, for any fingerprint bit $f,\text{Pr}\left(B=1,{𝒰}_{1}(s)\;\text{mod}\;\left\lfloor \frac{1}{2p}\right\rfloor =0\right)\geq \frac{1}{2}\times 2p=p$, which suggests that each ${\text{r}}_{i}[t,k]$ will be changed (i.e., XORed by 1) with probability higher than p, and this satisfies the sufficient condition developed in Theorem 1. □
